# Photo-oxidation of Micro- and Nanoplastics: Physical, Chemical, and Biological Effects in Environments

**DOI:** 10.1021/acs.est.3c07035

**Published:** 2024-01-03

**Authors:** Yanghui Xu, Qin Ou, Jan Peter van der Hoek, Gang Liu, Kim Maren Lompe

**Affiliations:** † Key Laboratory of Drinking Water Science and Technology, Research Centre for Eco-Environmental Sciences, Chinese Academy of Sciences, Beijing 100085, P. R. China; ‡ Section of Sanitary Engineering, Department of Water Management, Faculty of Civil Engineering and Geosciences, Delft University of Technology, Stevinweg 1, 2628 CN Delft, The Netherlands; § Waternet, Department Research & Innovation, P.O. Box 94370, 1090 GJ Amsterdam, The Netherlands; ∥ University of Chinese Academy of Sciences, Beijing 100049, P. R. China

**Keywords:** Microplastics, Nanoplastics, Photo-oxidation, Physical Effects, Photochemical Processes, Biological Effects

## Abstract

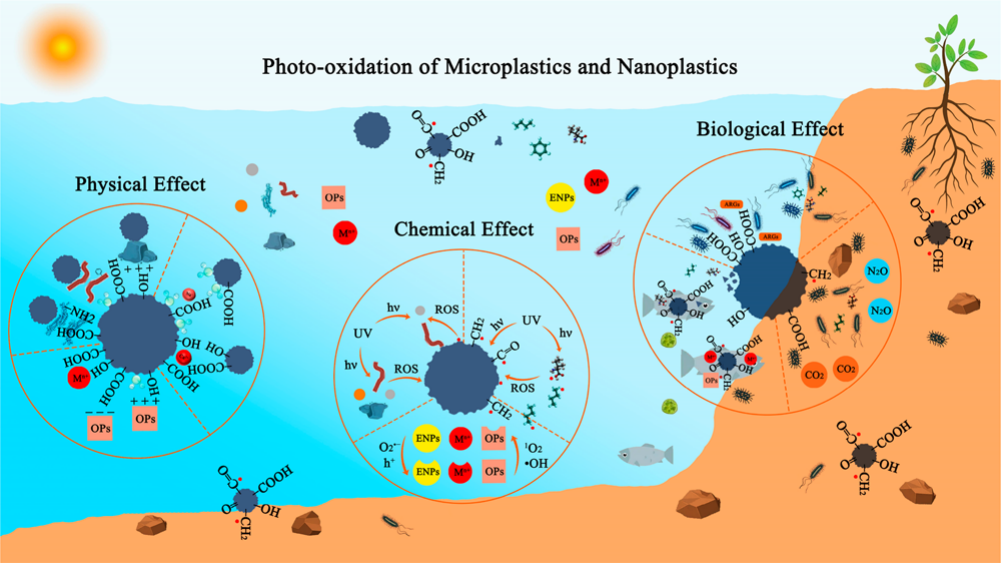

Micro- and nanoplastics (MNPs) are attracting increasing attention due to their persistence and potential ecological risks. This review critically summarizes the effects of photo-oxidation on the physical, chemical, and biological behaviors of MNPs in aquatic and terrestrial environments. The core of this paper explores how photo-oxidation-induced surface property changes in MNPs affect their adsorption toward contaminants, the stability and mobility of MNPs in water and porous media, as well as the transport of pollutants such as organic pollutants (OPs) and heavy metals (HMs). It then reviews the photochemical processes of MNPs with coexisting constituents, highlighting critical factors affecting the photo-oxidation of MNPs, and the contribution of MNPs to the phototransformation of other contaminants. The distinct biological effects and mechanism of aged MNPs are pointed out, in terms of the toxicity to aquatic organisms, biofilm formation, planktonic microbial growth, and soil and sediment microbial community and function. Furthermore, the research gaps and perspectives are put forward, regarding the underlying interaction mechanisms of MNPs with coexisting natural constituents and pollutants under photo-oxidation conditions, the combined effects of photo-oxidation and natural constituents on the fate of MNPs, and the microbiological effect of photoaged MNPs, especially the biotransformation of pollutants.

## 1 Introduction

With the high production and wide use of plastics, and the lack of effective waste disposal and recycling methods, plastics are increasingly accumulating in the environment.^1^ It is estimated that 19 to 23 million metric tons (11%) of global plastic entered aquatic ecosystems in 2016, and annual emissions may reach 53 million metric tons per year by 2030.^2^ In concordance with global production, polyethylene (PE), polyethylene terephthalate (PET), polypropylene (PP), polystyrene (PS), polyvinyl chloride (PVC), polyamide (PA) and polycarbonate (PC) are common polymers found in aquatic and terrestrial environments.^3−5^ In recent decades, small plastic particles called microplastics (MPs) (1 μm–5 mm in size) have attracted widespread attention in the world.^6^ A part of the MPs in the aquatic environment comes from the direct discharge of wastewater treatment plants and overland runoff, and the other part mainly comes from the mechanical, chemical, and/or biological degradation processes of large pieces of plastics.^7−12^ Nanoplastics (NPs) (<1 μm) are considered an extension of MPs but considerably differ from MPs in terms of transport characteristics, interactions with environmental constituents, bioavailability, and ecological risks.^13,14^

In the environment, MPs and NPs (MNPs) can undergo a series of weathering processes, mainly including mechanical fragmentation, photo-oxidation, thermal-degradation, and biodegradation.^15^ Photo-oxidation by sunlight is considered to be the most critical cause of polymer aging, which increasingly attracted attention over the past years.^16^ Up to March 2023, the total number of publications was 1892 based on the search results of the Web of Science with the following keywords: microplastic* or nanoplastic* and photo* or light* or ultraviolet* or UV*. The number of publications continuously increased from 68 in 2018 to 611 in 2022. Photo-oxidation of MNPs is a complex process involving free radicals such as alkyl, peroxyl, alkoxyl, and hydroxyl radicals (•OH),^17^ usually resulting in the change of physicochemical properties of MNPs, and inducing their fragmentation and leaching of organic matter like polymer molecules and additives.^18^ Photo-oxidation of MNPs in the environment is highly influenced by coexisting natural constituents of the surrounding matrix (e.g., ions, natural organic matter (NOM) and minerals).^19,20^ Notably, the presence of environmental constituents such as anions, cations, and minerals may accelerate the photo-oxidation of MNPs by promoting the generation of reactive oxygen species (ROS).^20−22^ However, some constituents like anions and NOM may inhibit the photo-oxidation of MNPs by either shielding light or scavenging ROS, although some debates exist.^23−25^

The modification of surface properties after photo-oxidation, such as increased O-containing functional groups and decreased hydrophobicity,^26^ can change the fate of MNPs in the environment.^27,28^ For instance, the effect of photo-oxidation generally increased colloidal stability and mobility of NPs in water and porous media,^28−30^ but this effect might be different depending on the water chemistry (e.g., salt types),^27,31^ the presence of NOM,^32,33^ and minerals in environments.^27,28,34^ As carriers of environmental contaminants, MNPs can adsorb the surrounding chemical substances and mediate their transport in the environment.^35^ The photo-oxidation of MNPs may enhance or reduce the adsorption capacity toward pollutants,^36^ and thus change the mobility of pollutants.^35,37^ Liu et al. reported that the photoaged PS NPs increased the mobility of both nonpolar (pyrene) and polar contaminants (4-nonylphenol) in saturated loamy sand compared to pristine NPs due to increased binding with contaminants.^37^ In addition to affecting the adsorption behavior of other contaminants, the photo-oxidation of MNPs may also produce environmentally persistent free radicals (EPFRs) and ROS, and mediate the photochemical transformation of pollutants, such as organic pollutants (OPs),^38,39^ heavy metals (HMs),^40^ and engineered nanoparticles (ENPs).^41,42^ The modified surface properties, the generated EPFRs and ROS, and the leached polymer molecules and additives of MNPs after photo-oxidation can also change their toxicities to organisms and affect microorganisms in the surrounding matrix, such as biofilm formation on MPs,^43^ the growth of planktonic microbes,^44,45^ as well as the microbial community in soil and sediment systems.^46,47^ All in all, photo-oxidation can influence the physical, chemical, and biological processes of MNPs in aquatic and terrestrial environments.

Currently, several reviews have summarized the weathering processes of MPs and corresponding effects on the environmental behavior of MPs, mainly considering the different weathering processes and mechanisms, and the effect of weathering on the properties, adsorption, and toxicity of MPs.^15,48−51^ However, few of them have thoroughly considered the physical, chemical, and biological effects of photo-oxidation of MNPs in the environment. In the last two years, an increasing number of studies have focused on understanding the process of photo-oxidation and its impact on different types of MNPs, as well as the effect of environmental constituents on this process.^20,22,24,52−54^ Furthermore, research on the influence of photo-oxidation of MNPs on the adsorption and photochemical transformation of other pollutants has gained more attention.^53−56^ The biological effect of photo-oxidized MNPs on organism activity, especially microbial community composition and function has also become a subject of interest among scholars.^43,45,57^ More importantly, the photo-oxidation process and the physical, chemical, and biological behavior of MNPs are mutually associated and inseparable. Thus, it is necessary to give a critical review of recent research on the physical, chemical, and biological effects of photoaged MNPs in aquatic and terrestrial environments.

First, this article introduces the photo-oxidation process and physicochemical changes of MNPs. Second, the physical effect of photo-oxidation of MNPs on the adsorption behavior toward pollutants, the colloidal stability of MNPs, as well as the transport behavior of MNPs and associated pollutants in porous media are discussed. Next, it dissects the effect of critical factors on the photo-oxidation of MNPs, and the mediated role of MNPs in the phototransformation of environmental pollutants. Moreover, this article assesses the effect of photo-oxidation of MNPs on the toxicity and its mechanism to organisms, plants and the microbiological effect on a variety of environmental microorganisms. Finally, knowledge gaps are pointed out and avenues for future research are suggested.

## 2 Photo-oxidation of MNPs

Sunlight irradiation is the most important aging process for MNPs in the environment.^58^ Sunlight is mainly composed of infrared (wavelength λ between 700 nm and 1 mm), visible (λ = 400–700 nm), and UV (λ = 100–400 nm).^59^ The UV fraction of light irradiation with high energy plays a critical role in the weathering of MNPs.^60^ It is estimated that the global average solar irradiance received on Earth is 16.4–34 mW/cm^2^ over a 24-h day.^61^ The photo-oxidation of MNPs has been extensively studied in experiments to accelerate aging with artificial light sources, mainly gas discharge lamps such as xenon, mercury, fluorescent and metal halide lamp, with UV intensities ranging from a few to tens of mW/cm^2^.^15,23,60,62^ The extent of photo-oxidation is determined by the UV dose (kJ/cm^2^) that is a product of solar irradiance (mW/cm^2^) and exposure time. Photo-oxidation experiments on MNPs are typically carried out in aqueous and aerial environments, commonly involving the participation of oxygen (O_2_) and water.^63^ Notably, in the laboratory, the mechanical agitation is typically combined with UV radiation to simulate hydraulic disturbance and to facilitate the mixing of samples. This can accelerate the photo-oxidation and disintegration to some extent by exposing the sample to more oxygen and UV radiation, as well as mechanical fragmentation.

The photo-oxidation first occurs on the surface of MNPs and produces highly reactive organic radicals and ROS that are involved in the radical reactions.^60^ In some cases, photo-oxidation can also lead to the formation of relatively stable EPFRs.^63^ Typically, along with the generation of alcohols, ketones, olefins, and aldehydes, carboxylic acids and eaters, vinyl groups and O-containing functional groups, such as carbonyl, carboxyl, and hydroxyl groups can be formed during the photo-oxidation of MNPs.^64^ The yellowing of the polymer is a typical sign of the generation of the chromophore.^65^ At the same time, significant changes in surface properties can be observed, including increased surface roughness, specific surface area (SSA), polarity, hydrophilicity, and negative charges.^15,35^ With further oxidation with time, it gradually develops to the inner layer.^66^ The molecular weight of the polymer is reduced, the original physical properties are modified, and the material becomes fragile and more prone to fragmentation. Finally, the photo-oxidation can result in the leaching of DOC and additives,^67^ and further mineralization.^68^

## 3 Physical Behavior of MNPs after Photo-oxidation

### 3.1 Adsorption of Contaminants

**Figure 1:**
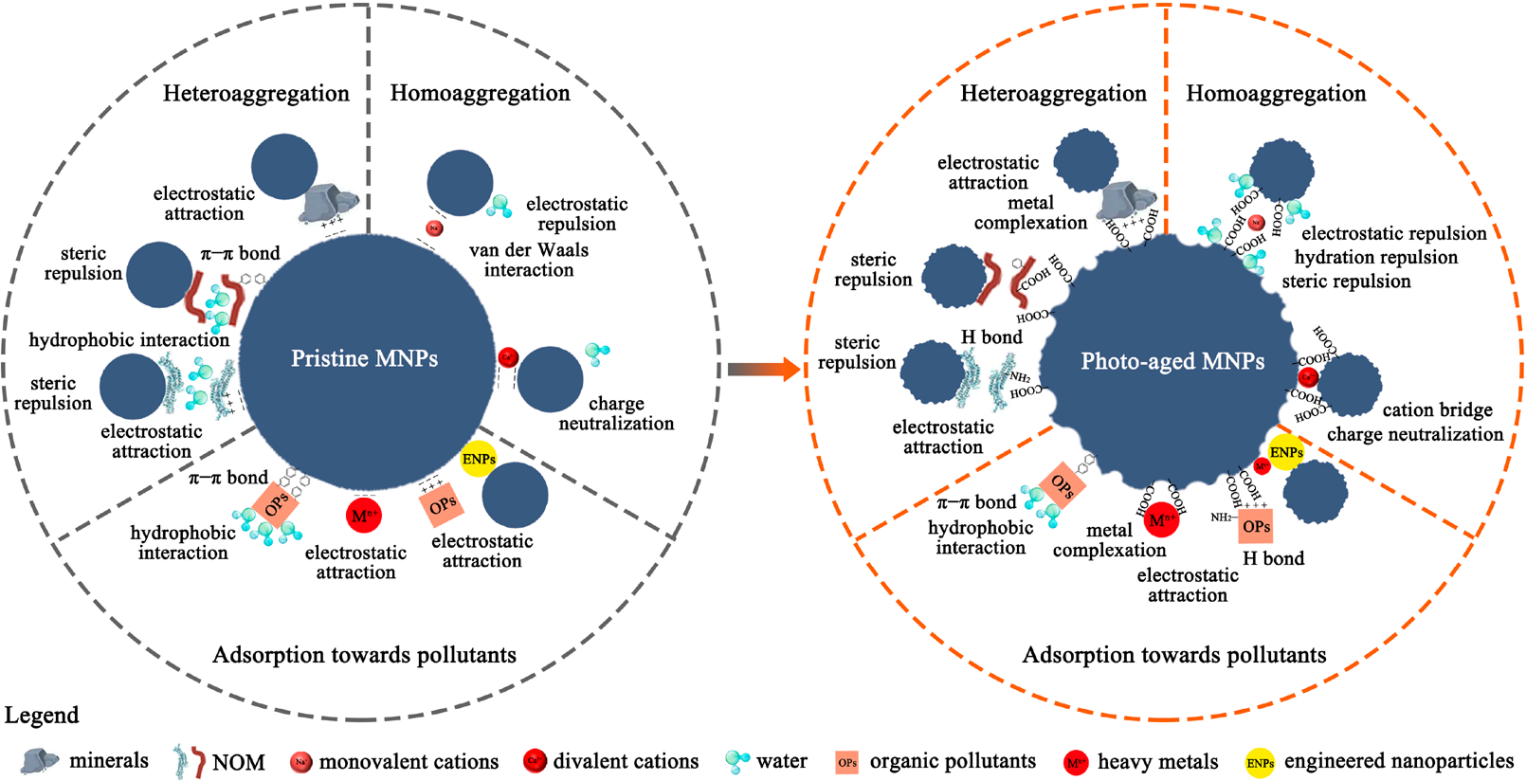
Effect of photo-oxidation of MNPs on the homoaggregation, heteroaggregation, and adsorption behaviors toward pollutants.

MNPs can act as vectors of environmental contaminants, such as OPs and HMs, and thus affect the transport of contaminants by MNPs in the environment and induce ecological risks.^7^ The effect of photo-oxidation on the adsorption behavior of MNPs is studied using batch adsorption experiments by determining adsorption kinetics and adsorption isotherms. The Langmuir and Freundlich models are the most commonly used models to describe adsorption of contaminants on pristine and aged MNPs, indicating mono- or multilayer adsorption processes on heterogeneous surfaces.^36,69,70^ The influence of photo-oxidation on the adsorption behavior of contaminants on MNPs mainly depends on three mechanisms (Figure 1): (1) the increase in surface roughness and SSA of aged MNPs could provide more adsorption sites for contaminants,^69,71^ (2) decreased hydrophobicity and increased O-containing functional groups can change adsorption affinity of contaminants to MNPs mediated by hydrophobic or hydrophilic interaction,^53,72^ and (3) increased electronegativity of aged MNPs could influence the electrostatic interaction between MNPs and charged OPs and HMs.^73,74^

#### 3.1.1 Organic Pollutants

Various forces including hydrophobic interaction, π–π bond, electrostatic interaction, and H-bond may mediate the interaction between MNPs and OPs (see Table S1 of the Supporting Information for details on the adsorption capacity and mechanism of photoaged MNPs toward OPs).^75^ The hydrophobicity of hydrophobic OPs can be evaluated with the partition coefficient of *n*-octanol/water (log *K*_OW_), which is linearly correlated with their adsorption on MNPs via hydrophobic interaction.^36,76^ Generally, due to the increase of O-containing functional groups, the hydrophobic interaction between MNPs and hydrophobic OPs will decrease with the photo-oxidation.^77^ π–π bond commonly occurs between aromatic MNPs (e.g., PS, PET, and PC) and OPs with C=C bonds or benzene rings.^69^ As typical aromatic polymers, photo-oxidation of PS MNPs can lead to a decrease in aromatic components and an increase in O-containing groups.^78^ Liu et al. reported that the adsorption of bisphenol A on aged PS MPs was reduced due to the decrease of the hydrophobic interaction and π–π bonds.^54^ For hydrophilic and polar OPs with abundant O- or N-containing groups, the H-bonds control their adsorption process on aged MNPs.^79^ Several studies reported that the adsorption ability of MNPs for hydrophilic compounds enhanced after photo-oxidation as they can form strong H-bonds.^73,75,80^ For example, Fan et al. found that the maximum adsorption capacity (*Q*_max_) of antibiotic on aged PLA and PVC MPs (1.29 kJ/cm^2^) were 1.2–2.2 times and 1.3–1.6 times higher than on the pristine MPs, respectively.^80^ For charged OPs, electrostatic interaction also affects their adsorption on MNPs.^73^ MNPs are commonly negatively charged under environmental pH conditions,^81^ and photo-oxidation could increase the negative charges of MNPs due to the increase in O-containing functional groups.^70^ Theoretically, photo-oxidation can decrease or increase the interaction between MNPs and negatively or positively charged OPs via electrostatic repulsion or attraction.^70^

However, the actual situations are more complex and depend on many factors such as the physicochemical properties of OPs, oxidation degree, and polymer type of MNPs. For example, the adsorption of benzalkonium chlorides (BAC) on pristine/photoaged MPs was related to the length of the saturated fat chains: the adsorption capacity of relatively hydrophobic BAC_14_ and BAC_16_ on aged PE MPs decreased by 19% compared with pristine PE MPs, but that of relatively hydrophilic BAC_12_ increased by 22% due to hydrophilic interaction and weak chemical interactions.^36^ Therefore, during photo-oxidation, the adsorption of OPs on MNPs is not determined by one force, but the result of a variety of forces. Liu et al. reported that hydrophobic and π–π interaction controlled the adsorption of atorvastatin and amlodipine on pristine PS MPs while electrostatic interaction and hydrogen bonding controlled their adsorption on aged PS MPs.^70^ Particularly, the adsorption capacity of atorvastatin on PS MPs decreased first and then increased with prolonged oxidation time, indicating the change of dominant forces.^70^ Thus, the UV dose also plays an important role in the adsorption of OPs on the surface of MNPs. In addition to oxidation time, the oxidation degree of MNPs could be affected by the light source and polymer type of MNPs. For instance, Liu et al. and Fan et al. reported that the adsorption capacity of ciprofloxacin (CIP) on aged MPs (e.g., PS, PVC, and PLA) was higher than on pristine MPs.^73,80^ Lin et al. found that the adsorption of CIP reduced on PS, PE, PET and PVC MPs after UV treatment, but increased on PVC MPs after vacuum UV treatment.^69^ The distinct adsorption phenomenon of CIP on aged PVC MPs in different publications might be related to different oxidation degrees of MPs and influenced by UV treatment time and polymer type. Similarly, Wang et al. suggested that the order of adsorption capacity of atrazine on pristine MPs was PS > PE > PP, while that on aged MPs was aged PE > aged PP > aged PS, which was explained by varying degrees of increase in surface roughness and SSA of aged MPs.^75^ Overall, photo-oxidation changes the adsorption of OPs on MNPs by influencing forces such as hydrophobic interaction and H-bond, which is also dependent on the oxidation degree and physicochemical properties of OPs.

#### 3.1.2 Heavy Metals

Different from OPs, photo-oxidation typically promotes the adsorption performance of MNPs to HMs.^16,71,72,82−84^ First of all, photo-oxidation can enhance the surface roughness and SSA of MNPs, and thus promote the adsorption of HMs.^16,84^ With increasing aging time, the surface of PS NPs significantly generated pores and became rough, and the adsorption of five heavy metal ions (Pb^2+^, Cu^2+^, Cd^2+^, Ni^2+^, and Zn^2+^) was enhanced.^16^ The increase in surface negative charges after UV irradiation also facilitates the adsorption of HMs due to enhanced electrostatic attraction. For example, the average zeta potential of tire wear particles (TWP) reduced from −8.0 to −14.6 mV and PP MPs from −5.4 to −9.5 mV after a UV dose of 0.86 kJ/cm^2^, and thus the orders of adsorption capacity of MPs toward Cd^2+^ and Pb^2+^ followed aged TWP > aged PP > pristine TWP > pristine PP.^82^ At the same time, the surface O-containing functional groups of aged MPs can strongly adsorb HMs via ion complexation.^71^

HMs do not exist alone and may coexist with other contaminants like OPs. Several studies also reported the synergistic or competitive adsorption of HMs and OPs on aged MNPs.^85,86^ Xue et al. suggested that the photo-oxidation promoted the coadsorption of Cu(II) and oxytetracycline (OTC) on thermoplastic polyurethanes (TPU) MPs, due to the increased surface roughness and functional groups.^86^ The synergistic effect was due to the bridging role of Cu(II) and the production of Cu(II)-OTC complex.^86^ Zhou et al. also reported that rough surface and O-containing functional groups on aged PS and PVC MPs were responsible for the adsorption of CIP and HMs, while CIP had negative and positive impacts on the adsorption of Cu(II) and Cr(VI) by aged MPs.^85^ The negative impact of CIP on the adsorption of Cu(II) may be due to the competitive adsorption and high steric hindrance effect, while nonspecific interactions between CIP-Cr(VI) complexes and the heterogeneous surface of aged MPs as well as CIP bridging promoted the adsorption of Cr(VI) on aged MPs.^85^

Generally, HMs are characterized by positive charges and high complexing capacity with negative charged groups while OPs exhibit complexity due to variations in hydrophobicity and charge. Therefore, the photo-oxidation typically increases the adsorption capacity of MNPs toward HMs via electrostatic attraction and ion complexation. However, the effect of photo-oxidation on the adsorption of OPs varies, influenced by diverse forces such as hydrophobic interaction, electrostatic interaction, π–π bonds, and H-bonds, depending on the polymer type and oxidation degree of MNPs and physicochemical properties of OPs.

### 3.2 Colloidal Stability in Water Media

Colloidal stability including homoaggregation (aggregation with themselves) and heteroaggregation (aggregation with other colloids) is critical to evaluate the fate, transport and potential toxicity of MNPs, especially NPs, in aquatic environments.^13,14^ Dynamic light scattering (DLS) is a widely used technique to determine the aggregation process and colloidal stability of MNPs by measuring the change in hydrodynamic size of MNPs over time. The initial stage of NP aggregation, characterized by an increase in aggregation rate with increasing ionic strength, is referred to as the reaction-limited stage.^87^ The subsequent stage that the aggregation rate of NPs reaches a maximum and remains constant as ionic strength is further increased is known as the diffusion-limited stage.^78^ The critical coagulation concentration (CCC) is the ionic strength at which the transition from the reaction-limited stage to the diffusion-limited stage occurs. CCC can be used as an indicator of the stability of NPs in solution, with higher CCC values indicating greater stability.^88^ By modifying the surface properties of NPs and affecting their interaction with environmental substances, photo-oxidation can also influence their homoaggregation and heteroaggregation behaviors (see Table S2 for details on the influence of photo-oxidation on the colloidal stability of NPs).

#### 3.2.1 Stability in the Presence of Monovalent and Divalent Cations

The photo-oxidation can influence the aggregation behavior of NPs by changing their interaction forces.^27,31^ Liu et al. reported that the CCC values of PS NPs exhibited a linear increase from 450 to 760 mM as the UV-radiation time was extended from 0 to 24 h (R^2^ = 0.975) in the presence of NaCl.^27^ Generally, the zeta potential of NPs became more negative with longer exposure to UV irradiation, which enhanced the electrostatic repulsion between NPs and colloidal stability of PS NPs in monovalent solutions.^27^ Mao et al. suggested that reduced hydrophobicity of aged PS NPs was responsible for the reduced aggregation due to enhanced hydration repulsion.^29,78^ That is, photoaged NPs contained more hydrophilic O-containing groups that easily form H bonds with water, and this hydration layer blocked the aggregation of NPs.^32,78^ In addition, photo-oxidized NPs could release organic molecules into solution, which may also induce the stabilization of PS NPs due to steric repulsion.^27^ Therefore, increased electrostatic repulsion, hydration force, and steric hindrance could explain the increased stability of NPs in the presence of monovalent cations after photo-oxidation. Cases are different in the presence of divalent cations. The O-containing functional groups of UV-irradiated NPs could bridge with Ca^2+^, which significantly reduced their stability in CaCl_2_ solutions.^27,32^ According to Liu et al., a negative linear correlation (R^2^ = 0.811) was observed between the CCC values and UV exposure time.^27^

In contrast, several studies concluded that photo-oxidation did not promote the aggregation of PS NPs in the presence of monovalent cations.^31,89^ Zhang et al. showed that simulated sunlight irradiation for 2 h exhibited a negligible effect on the aggregation of PS NPs.^89^ Wang et al. reported that pristine PS NPs are coated with sulfate groups, which were degraded first by photo-oxidation, thereby reducing the negative charges of PS NPs and enhancing their aggregation.^31^ The discrepancies may be attributed to differences in aggregation experiment design. Different from traditional aggregation studies, Zhang et al. studied the aggregation of NPs in phosphate buffer solutions (PBS, 1.0 mM) by observing the change in hydrodynamic size over 7 days in a oscillator.^89^ It might be difficult to observe a significant difference at low ionic strength due to the high stability of PS NPs.^31^ Wang et al. studied the aggregation of NPs under low-intensity UV exposure (0.0007 kJ/cm^2^), which induced slower polymer oxidation than in other studies.^27,32^ Therefore, the stability of PS NPs may first decrease and then increase in monovalent cationic solutions with the destruction of sulfate groups and subsequent generation of O-containing groups during the photo-oxidation process.^27,31^ Similarly, the decreased stability of PS NPs-NH_2_ was also observed after photo-oxidation due to the first oxidation of surface sulfate and amine groups.^31^ Thus, it is essential to take into account the impact of UV dose that may affect the outcome when designing relevant experiments. Notably, Zhang et al. observed that the hydrodynamic size of pristine PS NPs increased from 99 to 299, 444, and 833 nm after photo-oxidation under UV dose of 39.5, 78.9, and 157.8 kJ/cm^2^, respectively, which was explained by the cross-linking of PS^•^ or PSOO^•^ and subsequent production of PS–PS or PSOOPS.^90^ As reported, if O_2_ content is insufficient to react with these radicals, cross-linking reactions are more likely to occur instead of direct chain scission.^91^ Given that the details of the aging experiments, such as the concentrations of dissolved O_2_ and PS NPs, were not clearly defined,^90^ further investigation is necessary to determine whether the cross-linking of PS NPs could occur and lead to the aggregation of NPs under UV exposure.

In addition, several studies compared the aggregation behavior of normal PS NPs with carboxyl-functionalized PS NPs (PS-COOH) that may simulate surface properties of photo-oxidized PS NPs to some extent.^88,92^ However, the reported CCC values of PS-COOH in NaCl solutions were not higher than those of bare PS NPs (191 mM vs 264 mM, and 308 mM vs 310 mM), suggesting that PS-COOH may exhibit lower stability.^88,92^ Therefore, although conducting experiments with PS-COOH can be useful in exploring relevant mechanisms, it is important to recognize that these particles may differ from UV-aged NPs in important ways. First, UV exposure can induce changes in the physicochemical properties of PS NPs that may not be fully replicated by carboxyl-functionalization alone. Second, commercial PS and PS-COOH particles may be manufactured differently, which can make direct comparisons between the two difficult or unreliable.

#### 3.2.2 Stability in the Presence of Natural Colloids

In addition to influencing the homoaggregation of individual NPs, photo-oxidation can also affect the interaction or heteroaggregation of NPs with other environmental colloids. Adsorption of environmental and biological macromolecules on NP surfaces is well-studied to enhance the stability of NPs in monovalent cationic solutions due to steric repulsion.^87,88^ The photo-oxidation of NPs were reported to reduce their adsorption capacity toward organic molecules (e.g., HA, lysozyme, and alginate) and the thickness of adsorption layer, thereby decreasing the inhibitory effect of steric repulsion on subsequent aggregation of NPs.^32,33,78^ However, as opposed to HA, the inhibitory effect of bovine serum albumin (BSA) on the aggregation of PS NPs was strengthened after photo-oxidation due to stronger hydrogen bonding and electrostatic attraction between O-containing functional groups on aged NPs with amide groups of BSA.^33^ Dissolved black carbon (DBC) was reported to enhance the aggregation of PS NPs in monovalent cationic solutions as the strong interaction between aromatic constituents of DBC and PS NPs partially screened negative charges of PS NPs; the photo-oxidation decreased the interaction between DBC and PS NPs, and decreased the promoting effect of DBC on the aggregation of PS NPs.^78^

Photo-oxidation can also alter the interaction between natural minerals and NPs, affecting their aggregation and sedimentation in environments. Zhang et al. suggested that positively charged iron oxides (e.g., goethite and magnetite) showed stronger interactions with aged PS NPs than pristine PS NPs due to increased electrostatic attraction and ligand exchange.^34^ Although the aged PS NPs were more negatively charged than pristine PS NPs, the enhanced adsorption of aged PS NPs on negatively charged clay minerals (e.g., kaolinite and montmorillonite) was also observed, which was attributed to strong ligand exchange between O-containing functional groups with hydroxyl groups on mineral surfaces.^34,93,94^ Consequently, the stronger interaction between aged PS NPs and minerals compared to pristine NPs, makes the aged NPs more susceptible to heteroaggregation, adsorption, and coprecipitation with the minerals.^34^ Therefore, the photo-oxidation of NPs can enhance or reduce the interaction or heteroaggregation with natural colloids depending on distinct interfacial interaction, and further affect the aggregation of NPs in complex water media.

However, NPs were unlikely to undergo photo-oxidation alone, as the coexisting natural substances might take part in the photo-oxidation of NPs complex systems. These natural colloids may undergo phototransformation or affect the photo-oxidation of NPs, and subsequently affect the stability of NPs. For example, HA might compete with NPs for photons and undergo photodegradation, and the destruction of adsorbed HA increased (in NaCl) or decreased (in CaCl_2_) the aggregation of NPs.^33^ However, light irradiation induced the flocculation of BSA molecules that wrapped and integrated NPs, resulting in the formation of large aggregates.^33^ Giri et al. observed a significant increase in the hydrodynamic diameter when NPs were photo-oxidized (48 h) with microalgae extracellular polymeric substances (EPS), compared to the case that NPs were aged in the lake water medium alone (without EPS), which was explained by the formation of EPS layer on NPs during the photo-oxidation process.^95^ Although the mechanism was not mentioned, the increased particle size might contribute to the heteroaggregation between NPs and photoflocculated EPS.^33,96^

### 3.3 Transport Behavior in Porous Media

Soil and sediment are not only a sink of MNPs, but may also represent potential sources of MNPs pollution in groundwater systems.^35,97,98^ The transport and deposition process of MNPs in soil and sediment were commonly studied using porous media transport experiments and quartz crystal microbalance with dissipation (QCM-D).^99−102^ Although no studies have reported this, photo-oxidation of MNPs may decrease the particle size and thus increase its migration into the pore throat of soil and sediment media. Photo-oxidation can also influence the mobility of MNPs in porous media by changing the surface properties of MNPs and hence the interaction between MNPs and soil media.^28,37,78^ In addition, MNPs in soils are likely to adsorb a variety of contaminants such as OPs, HMs, and ENPs, and affect their transport in soils.^7,103,104^ As reviewed above, by changing their adsorption capacity toward these contaminants, photo-oxidation of MNPs might increase or reduce the mobility of contaminants.

#### 3.3.1 Enhanced Mobility of MNPs

Typically, photo-oxidation can increase the mobility of MNPs in porous media.^28,37^ Consistent with the predictions of the classic Derjaguin–Landau–Verwey–Overbeek (DLVO) theory, Ren et al. demonstrated that photoaged PS MPs displayed increased mobility in both sandy and clay loam soils, attributed to a more negative charge compared to pristine MPs.^28^ The widely used DLVO theory that takes into consideration van der Waals forces and electrostatic interactions, is instrumental in predicting the mobility of charged ENPs in porous media.^105,106^ Unlike common hydrophilic ENPs, such as ZnO,^106^ TiO_2_,^106^ and graphene oxide,^107^ MNPs exhibit hydrophobic characteristics, and photo-oxidation generally leads to a reduction in their hydrophobic nature.^18,26^ In certain cases, the DLVO theory fails to accurately predict the mobility of MNPs in porous media.^37,108^ Liu et al. indicated that the contribution from increased negative charge was relatively small, whereas photoaging-induced increase in hydrophilicity was the primary cause for the enhanced mobility of PS NPs.^37^ Thus, the DLVO theory was less suitable to explain the transport behavior of PS NPs than the extended DLVO (XDLVO) that considers the hydrophobic effect.^37^ Feng et al. observed contrasting effects of photo-oxidation on the transport of two MPs (PE and PTFE) in shore substrates over tidal cycles.^108^ Aged PE MPs that were more negatively charged and more hydrophilic compared to pristine MPs, demonstrated greater transport in porous media, aligning with the predictions of both the DLVO and XDLVO theory.^108^ Conversely, aged PTFE MPs exhibited enhanced retention in porous media despite a decline in negative charges.^108^ This discrepancy was explained with increased surface roughness of aged PTFE MPs,^108^ but the notable increase in hydrophobicity also suggests that hydrophobic effects might contribute as another potential cause.^37^ Hence, although surface charge plays a role, the alteration in other polymer properties after photo-oxidation, particularly hydrophobicity, is of crucial importance. It is essential to consider other non-DLVO interactions when assessing the impact of photo-oxidation on the mobility of MNPs.

The presence of other environmental substances also may affect the mobility of aged MNPs in porous media by influencing the interaction between MNPs and media. The presence of HA in either solution or silica surface inhibited the deposition of PS NPs on the silica surface mainly due to additional steric repulsion, but this inhibitory effect was weakened after the photo-oxidation of PS NPs.^78^ Similarly, photo-oxidation also reduced the promoting effect of DBC on the deposition behavior of PS NPs on silica surfaces due to the weak interaction between DBC and aged PS NPs.^78^ The positively charged Fe minerals may enhance the retention of aged MNPs in porous media due to electrostatic attraction and complexation,^28,34^ but the actual effect may be related to the content of minerals and the aging degree of MNPs. Although current studies provided important information on the possible effect of natural colloids on the transport of pristine and aged MNPs in porous media, it is still limited to understand the combined effect of photo-oxidation and natural colloids such as NOM, minerals, bacteria, and biofilms on the mobility of MNPs in porous media.

#### 3.3.2 Mediated Transport of Contaminants

MNPs in soils are likely to adsorb a variety of contaminants such as OPs, HMs, and ENPs, and affect their transport in soils.^7,103,104^ As reviewed above, by altering their adsorption capacity toward contaminants, photo-oxidation of MNPs might alter the mobility of contaminants in porous media. The effects may be distinct for two different cases: (i) MNPs and contaminants coexisted in aquatic media and cotransported, and (ii) MNPs were retained in soil media followed by the introduction of contaminants. In terms of the cotransport case, Liu et al. reported that the photo-oxidation of PS NPs increased the contaminant-mobilizing ability of PS NPs in saturated loamy sand due to increased binding with both nonpolar (pyrene) and polar contaminants (4-nonylphenol).^37^ Considering that photo-oxidation can promote or inhibit the adsorption of OPs depending on various factors, the mobility of OPs may be enhanced or reduced after the photo-oxidation of MNPs, although limited studies have investigated this. Similarly, due to the increase in O-containing functional groups, the promotion effect of UV-aged PS NPs on the transport of Pb(II) and Cd(II) was stronger than that of the pristine NPs.^30^ Furthermore, as carriers, aged PS NPs were more capable of freeing HMs retained in porous media.^30^

For the case that MNPs were predeposited on soil media, Hu et al. found that UV irradiation of PS, PVC, and PE MPs enhanced their positive effect on the adsorption of 17β-estradiol in soil, indicating that the input of aged MPs into soil might reduce the mobility of 17β-estradiol by enhancing the adsorption capacity of the soil.^109^ Similarly, after being incubated with sediments, aged PE MPs also showed higher retention capacity toward Pb(II) than pristine PE MPs due to enhanced electrostatic attraction.^83^ However, photo-oxidation of PLA MPs might increase the mobility of Pb(II) in sediments as aged PLA MPs changed the microbial community in sediments and further altered the zeta potential of the mixture of MPs and sediments.^83^ Therefore, the effect of photo-oxidation of MNPs on the mobility of contaminants in porous media may be complex, depending on factors such as transport mode, polymer type, size and oxidation degree of MNPs, soil properties, and indirect factors like biological effects.

## 4 Photochemical Processes with Coexisting Constituents

**Figure 2:**
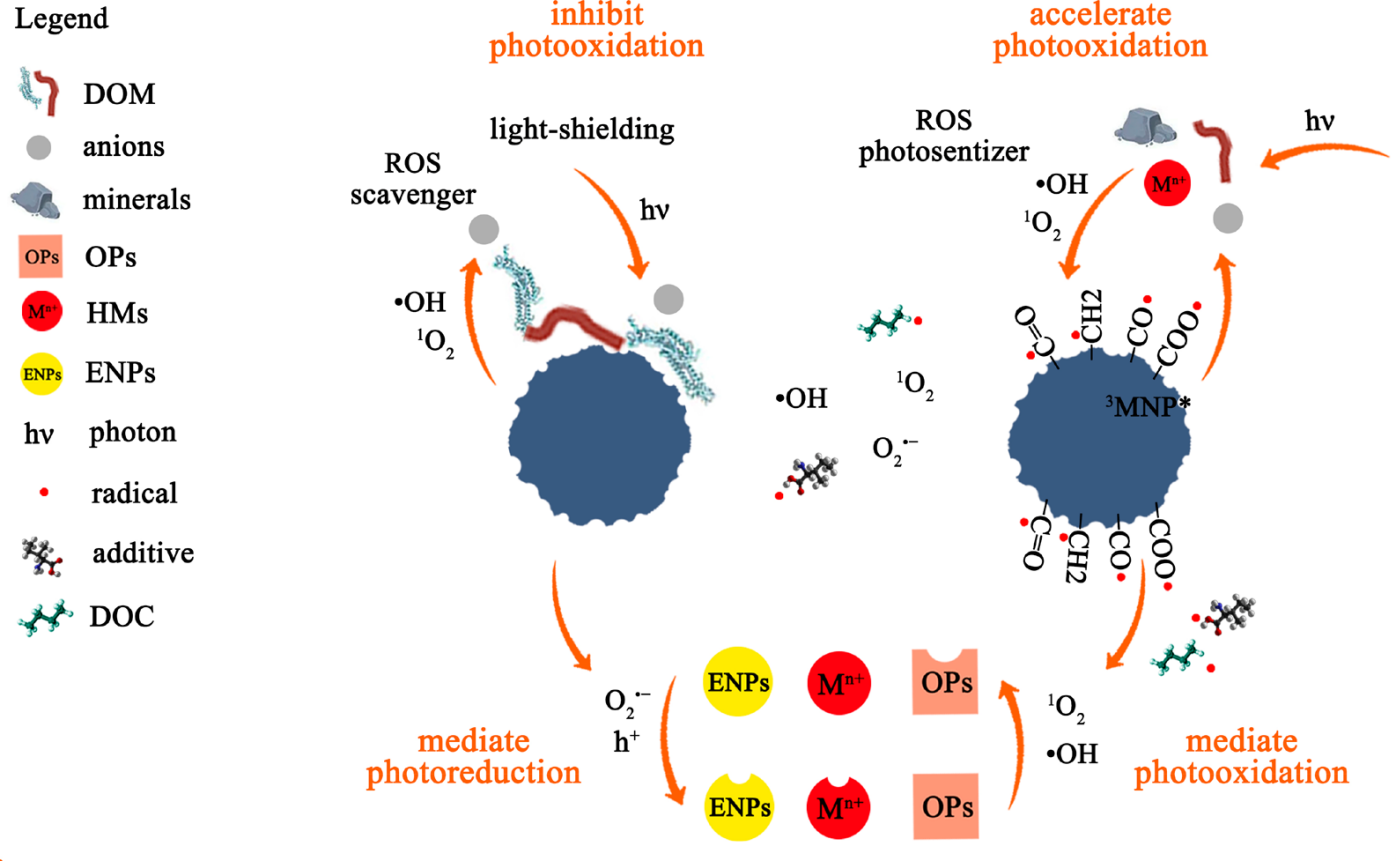
Photochemical processes of MNPs with coexisting natural constituents and pollutants.

The photo-oxidation of MNPs involves the direct absorption of specific wavelengths of light energy, leading to the generation of excited states of electrons and alkyl radicals.^60^ As the process progresses, produced highly reactive organic radicals such as alkyl, peroxyl, and alkoxyl radicals can facilitate self-catalyzed reactions.^60,110^ In particular, the generation of •OH with strong oxidation potential and high electrophilicity further accelerates the photo-oxidation of MNPs.^17^ In addition, aromatic MNPs (e.g., PS, PET and PC) are capable of producing an excited triplet state (^3^MNP*) upon exposure to UV radiation.^111^ The ^3^MNP* can transfer energy to dissolved O_2_ and water molecules to produce other ROS such as ^1^O_2_ and O_2_^•–^, accelerating photo-oxidation of aromatic MNPs.^26,111,112^ However, in the real environments, MNPs do not undergo photo-oxidation in isolation but rather in the presence of various environmental components, such as inorganic ions, natural colloids, and dissolved organic matter (DOM). Under UV irradiation, these coexisting components can also undergo photochemical processes, absorbing photons and either consuming or producing ROS.^25,113^ For example, the abundant chromophores in DOM can absorb UV energy, which forms higher energy excited states (^3^DOM*) that can initiate reactions with dissolved oxygen and water molecules, resulting in the production of ROS through energy transfer.^114,115^ Mediating by these photochemical processes, the coexisting components can either accelerate or inhibit the photo-oxidation process of MNPs, as illustrated in Table S3 (highlighting critical factors influencing the photo-oxidation of MNPs) and Figure 2.

### 4.1 Effects of Natural Substances on the Photo-Oxidation of MNPs

Inorganic ions in aquatic environments may influence UV penetration depth and free radical reactions, and thus induce a different degree of photo-oxidation of MNPs.^116,117^ Some studies found that MPs (e.g., PP, PE, and PS) were degraded more efficiently in ultrapure water compared with seawater due to high salt concentrations induce the high refractive index of water and salt crystals formed on MP surfaces.^116,117^ The role of inorganic ions in free radical reactions is still of a matter of dispute. Studies indicated that Cl^–^ can effectively capture •OH radicals and inhibit the formation of O_2_^•–^, thus weakening the role of ROS in the photo-oxidation of MPs (e.g., PVC and PP).^19,25^ However, Zhu et al. reported that NO_3_^–^, Br^–^, and Cl^–^ accelerated the indirect photo-oxidation of PS MPs by reacting with ^3^PS* to promote the formation of reactive halide radicals and •OH; although HCO_3_^–^ scavenged •OH, HCO_3_^–^ had no inhibitory effect on PS aging due to the oxidation role of generated CO_3_^•–^.^22^ The distinct roles of halide ions in the photo-oxidation of MPs may be related to different polymer types. Halide ions exhibit ROS-scavenging effects that inhibit the photo-oxidation of aliphatic MNPs (e.g., PP, PE, and PVC).^19,25^ In contrast, for aromatic MNPs (e.g., PS, PET and PC), halide ions can react with the excited state ^3^MNP*, generating highly reactive halide radicals that promote photo-oxidation and enhance the production of •OH.^22^

As the main inorganic colloidal component in aquatic environments, natural minerals may take part in the photo-oxidation process of MPs. All reported minerals including kaolinite, montmorillonite, goethite, hematite, and pyrite can promote the photo-oxidation of MPs,^20,52,118^ but mechanisms are distinct. The presence of kaolinite and montmorillonite could stabilize the MP radical cations and prevent their recombination with hydrated electrons, thus promoting the generation of •OH and photodegradation of PVC and PET MPs.^20^ Under UV irradiation, the surface Fe(II) phases of goethite and hematite could catalyze the generation of H_2_O_2_ and Fe^2+^, leading to the initiation of the light-driven Fenton reaction.^52^ This process produced a large amount of •OH and accelerated the photo-oxidation of PP and PE MPs.^52^ Similarly, the photo-oxidation of PS MPs and the transformation of intermediates were accelerated in the presence of pyrite due to the generation of ROS, especially •OH.^118^

DOM which contains chromophores such as carbonyl, carboxyl, hydroxyl, and benzene rings, serves as important photosensitizers in natural waters.^119,120^ There is a dispute regarding the influence of DOM on the photo-oxidation of MNPs. As both ROS scavengers (e.g., •OH and O_2_^•–^) and optical light filters, humic acid (HA) and fulvic acid (FA) were reported to inhibit the photo-oxidation of PP MPs.^24^ The aging process of PS MPs was accelerated in the presence of HA and FA,^19,89^ and FA exhibited a more significant promoting effect than HA due to their more active carboxyl structure that produce more •OH.^121^ However, Cao et al. indicated that HA/FA accelerated the photoaging of aliphatic PP MPs due to the generation of •OH by DOM photosensitization, while it inhibited or had only a minor effect on the photo-oxidation of aromatic PS and PC MPs/NPs.^122^ The explanation provided was that PS NPs with large SSA can adsorb sufficient DOM via π–π interactions, delaying photoaging by competing for photon absorption sites, while released phenolic compounds from aromatic MPs weaken the photoaging process by quenching •OH.^122^ While largely unclear, the varying impacts of DOM on the photo-oxidation of MNPs might be related to DOM photosensitization and light shielding ability, which can be influenced by molecular characteristics of DOM and adsorption extent of DOM on MNPs. Therefore, the physicochemical properties of MNPs (e.g., size, type, hydrophobicity, and crystal structure) and DOM (e.g., molecular weight distribution, hydrophobicity and functional groups) potentially determine the role of DOM in the photo-oxidation of MNPs.

Therefore, MNPs not only undergo the photo-oxidation process but also interact with a wide range of environmental components that may affect their photoreactivity in different ways. Depending on their specific roles, these constituents may either act as inhibitors, impeding the photo-oxidation of MNPs, or promoters, accelerating it. The dual nature of their impact is contingent on whether they function as scavengers, competing for photons or ROS, or as photosensitizers, actively promoting the generation of ROS during the photo-oxidation of MNPs. This intricate interplay underscores the complexity of the environmental factors that modulate the photodegradation of MNPs. Notably, most studies examined the photo-oxidation of MNPs under controlled conditions that can provide valuable insights into the mechanisms involved, but it may not fully reflect the complex interactions and phototransformations that occur in real-world environments.

### 4.2 Effects of MNPs on the Photochemical Transformations of Other Contaminants

When exposed to sunlight, OPs can undergo spontaneous phototransformation, representing an important process of their attenuation in natural waters.^123^ The mechanism is direct photolysis and indirect phototransformation induced by environmental components, such as anions, cations, and DOM. The reactive intermediates such as ^3^DOM* and ROS generated from this photosensitization process can participate in the photodegradation of OPs.^124−126^ On the one hand, MNPs that usually show good optical absorption characteristics may compete for photons with OPs and thus inhibit the direct photolysis of OPs due to the light screening/shielding (see Table S4 for details on the role of MNPs in the phototransformation of pollutants).^38,56,127,128^ On the other hand, MNPs can also take part in the indirect phototransformation of OPs by generating ESPRs, ROS, and ^3^MNPs* and by providing more surfaces for pollutant adsorption and photoreactions (Table S4). For example, Zhang et al. reported that the degradation of sulfamethoxazole (SMX) was decreased in the presence PS MPs and even more so in the presence of aged PS MPs,^38^ as the optical absorption capacity of PS MPs gradually increased with the photoaging time after the formation of new chromophores, such as carbonyl groups and conjugated double bonds.^38^ Additional studies also noted that the presence of photoaged MNPs resulted in decreased degradation of OPs compared to pristine ones.^39,56,128,129^ However, most studies reported that MNPs had a promoting effect on the photo-oxidation of OPs.^55,128,130−133^ Wang et al. indicated that the photodegradation efficiency of atorvastatin (ATV) increased from 19.82% to 50.27% in the presence of 0.01 g/L and 0.5 g/L PS MPs, respectively.^132^^1^O_2_ generated from photosensitization of PS MPs was the main reason; besides, the role of ^3^PS* became important in the presence of aged MPs because ^1^O_2_ can be generated from the ^3^PS*.^132^ The light-screening and photosensitization effects induced by MNPs can occur simultaneously, where the former inhibits the direct photodegradation of pollutants, while the latter promotes indirect photodegradation.^127^ The MNPs-mediated phototransformation of OPs may differ depending on the properties of OPs. Wang et al. reported that the catalytic effect of MPs was strongly dependent on the electron-donating ability of functional groups of OPs.^133^ Additionally, different types of MNPs also have a different effect on the phototransformation of pollutants. The photolysis of 2,2′,4,4′-tetrabromodiphenyl ether was inhibited by aged PS MPs compared with pristine ones as the aged PS caused more light-shielding effect, while aged PP and PE increased pollutant degradation because the fragmentation of aged MPs provided more contact surfaces for pollutants and light.^56^

The photo-oxidation of MNPs can also mediate the oxidation or reduction of HMs (Table S4). Up to now, limited publications have studied the phototransformation of HMs in the presence of MPs. On the one hand, the photoreduction of HMs (e.g., Cr(VI) and Ag(I)) was accelerated in the presence of MNPs by processes such as O_2_^•–^ production,^40,134,135^ or electron shutting by carbonyl groups on oxidized MPs.^135^ On the other hand, the photo-oxidation of certain HMs such as Mn(II) can be accelerated in the presence of MNPs due to the generation of ROO^•^ and O_2_^•–^ radicals.^136^ The photo-oxidation of MNPs can also induce the dissolution of ENPs, such as nano-Ag and nano-ZnO,^41,42^ due to the generation of ^1^O_2_, •OH, and/or acid release.^42^ Simultaneously, reductive O_2_^•–^ could also reduce the released Ag(I) to secondary nano-Ag.^42^ It is important to note that the role of photo-oxidation in the transformation of metal and metalloid species is reversible and interchangeable, likely depending on the redox potential of the system. These findings highlight the complex interplay between photo-oxidation processes and the fate of MNPs and associated contaminants in environmental systems.

## 5 Biological Effects of Photo-Aged MNPs

### 5.1 Toxicity to Aquatic Organisms

#### 5.1.1 Enhanced or Reduced Toxicity of MNPs

**Figure 3:**
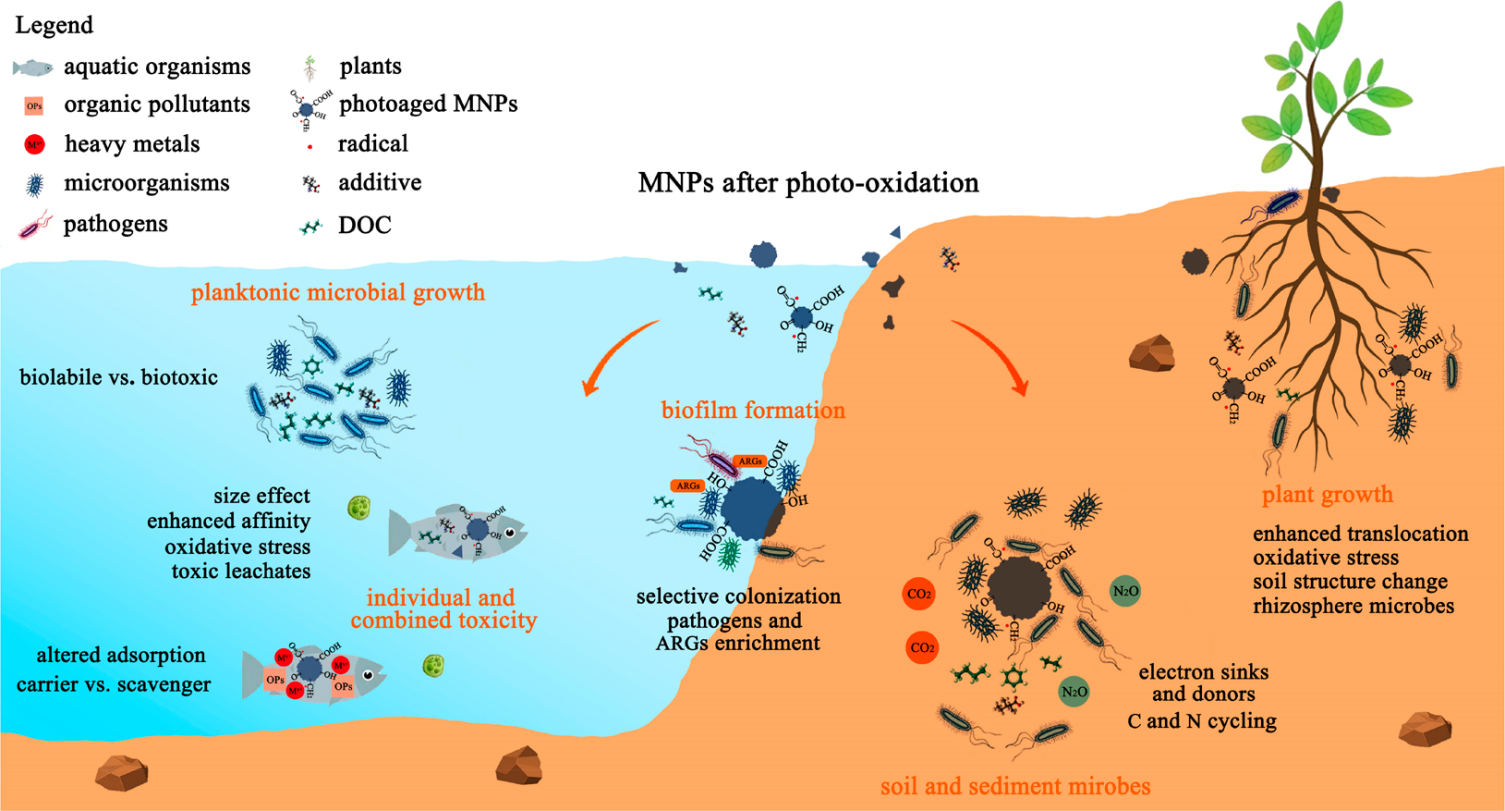
Biological effects of MNPs after photo-oxidation.

The toxicity of MNPs to aquatic organisms in environments can be changed after the photo-oxidation (see Figure 3 and Table S5 for details on the biological effects of photo-oxidized MNPs on organisms and microbes). The majority of the studies (6 out of 8 publications) reported that the photoaging of MNPs can enhance their biological toxicity, mainly originating from four reasons: (i) the fragmentation of MNPs after photodegradation can generate smaller-sized particles with an irregular shape, which enhance the toxicity of MNPs to organisms due to size and surface area effects. These small particles can be ingested and bioaccumulated in organisms through sorption, endocytosis, and phagocytosis compared to virgin MPs.^49,137,138^ In addition, these fragments with larger surface roughness and surface-specific area show higher affinity to the tissues and cells and restrained the nutrients absorbed into the cells.^138,139^ (ii) The photoaging of MNPs can enhance the surface O-containing functional groups and negative charges, which may increase the interaction between MPs and proteins in biota via hydrogen bonding and electrostatic interaction.^139,140^ (iii) Aged MNPs can also result in more oxidative stress due to the generation of EPFRs and ROS,^141−143^ which led to higher cytotoxicity compared to pristine ones.^144^ (iv) The enhanced toxicity of photoaged MNPs to organisms may be attributed to the leachates including additives and MNP-derived intermediates.^145−147^ The bioaccumulation of the leached endogenous toxicants (mainly phthalates) from photodegraded PS MPs contributed to the exacerbated hepatotoxicity of Grouper.^142^ Luo et al. demonstrated that longer radiation time led to more release of Cr and Pb from commercial lead chromate pigmented MPs, exhibiting more inhibitory effects on the cell growth and photosynthesis of *Microcystis aeruginosa*.^148^

However, few studies concluded the opposite results that photo-oxidation could alleviate the toxicity of MNPs to organisms.^95,141^ Zou et al. indicated that pristine small-sized PA MPs (8.13 μm) with a high aggregation potential tended to accumulate in organisms, while photoaged ones with higher stability were more easily to be excreted by zebrafish larvae.^141^ Such a distinct result may be also related to the larger particle size of pristine MPs, which were easily intercepted by the intestinal villi and difficult to be excreted directly by the larvae.^137^ However, Giri et al. found that the UV radiation significantly enhanced the hydrodynamic size of PS NPs in media containing EPS due to photoinduced agglomeration of PS NPs and EPS, and this mitigated the toxic effects of PS NPs on freshwater microalgae.^95^

Therefore, size and surface area effects, the formation of O-containing groups, the generation of EPFRs, and the leachates from photo-oxidized MNPs are responsible for their higher biotoxicity compared to pristine ones but there are cases where size changes explain the lower toxicity of photoaged MNPs. In real environments, the biotoxicity change of aged MNPs may result from a combination of multiple causes,^137,138,142^ and can also be affected by the coexisting environmental substrates.

#### 5.1.2 Combined Toxicity with Environmental Contaminants

As vectors for environmental contaminants, MNPs can affect their migration and bioaccumulation in organisms via ingestion.^149,150^ The photo-oxidation can change the interaction between MNPs and contaminants by surface modification, which may further affect their combined risk in environments. Several studies have investigated the effect of photo-oxidation on the combined toxicity of MNPs and pollutants, such as HMs, OPs, and ENPs to organisms. Studies indicated that aged MPs can decrease the bioavailability of coexisting HMs (Cu^2+^ and Cd^2+^) to microalgae *C. vulgaris* due to their strong adsorption capacity toward HMs.^140,151^ Another reason for decreased bioavailability may be that photo-oxidation increased the aggregation and sedimentation of MPs with HMs, and reduced the concentration of HMs and MPs in the aquatic phase, thus limiting their inhibitory effect on organisms.^151,152^ The opposite results were reported in terms of the combined toxicity of MNPs and metal nanoparticles, such as nano-ZnO and nano-Ag.^41,42^ PS MNPs increased the sunlight-induced dissolution of nanoparticles, and served as vectors for dissolved metal ions, significantly increasing the ion-related toxicity of nanoparticles.^41,42^

However, cases can be complicated for the combined effect of OPs and MNPs after photo-oxidation due to the different role of MNPs (carrier vs scavenger) in the bioaccumulation of OPs, as well as possible increase or decrease in the adsorption capacity of aged MNPs toward OPs.^69,73,80^ On the one hand, MNPs can facilitate the mobility of OPs, and photo-oxidation enhances or reduces the bioaccumulation of OPs depending on the changed adsorption capacity.^153,154^ For example, the stress of tilapia caused by PS MPs and propranolol (PRP) was alleviated while those by MPs and sulfamethoxazole (SMX) were exacerbated after photo-oxidation of MPs, as aged MPs adsorbed less PRP but more SMX compared to pristine ones.^154^ On the other hand, pristine or aged MNPs may serve as a scavenger for OPs, reducing their biouptake.^155^ Kim et al. suggested that the aged PE MPs reduced the toxicity of BAC_12_ but enhanced the toxicity of BAC_16_ as the adsorption of BAC_12_ was increased but that of relatively hydrophobic BAC_16_ was decreased after the aging of MPs.^36^

Therefore, the effect of photo-oxidation on the combined toxicity of MNPs and contaminants depends on both the roles of MNPs as carrier or scavenger and the adsorption capacity of pristine/photoaged MNPs. If MNPs act as carriers, the toxicity resulting from the combined effect of MNPs and contaminants is positively linked to the contaminants’ adsorption capacity on pristine/photoaged MNPs. Conversely, if MNPs act as scavengers, the combined toxicity is inversely related to the contaminants’ adsorption capacity on pristine/photoaged MNPs. However, whether and how photo-oxidation can affect the combined toxicity of MNPs and contaminants needs to be studied in more detail. Current studies lack a consistent approach to assess these effects, and the results may be distinct given the polymer type, size, and the aging degree of MNPs. Moreover, in addition to altering the interaction between MNPs and contaminants and bioaccumulation, photo-oxidation may also affect the chemical transformation of these contaminants. Thus, knowledge regarding the possible role of photochemical effects is needed to understand the ecological risks of coexisted MPs and pollutants in the environment.

### 5.2 Effects on Microorganisms

#### 5.2.1 Biofilm Formation

Biofilm can be formed on the surface of MPs due to the accumulation of a large number of microbial communities in the aquatic environment.^8,156^ MP biofilm is distinctive and considered a new ecological niche, influenced by MP surface characteristics and the surrounding environmental matrix.^157,158^ As an important process modifying the surface properties of MPs, photo-oxidation has been studied to affect the formation of biofilm and microbial community composition. Due to the increase in surface roughness and SSA, aged MPs were more conducive to microbial adhesion.^159,160^ The formation of O-containing groups and increased hydrophilicity of MPs after photo-oxidation also may select hydrophilic microorganisms to colonize.^43^ Additionally, the leaching of degraded polymer from photoaged MPs as a carbon source, as well as the enhanced adsorption of nutrients, may affect the colonization of microorganisms.^8,157^ Studies indicated that the total biomass, detected operational taxonomic unit (OTU) number, and α diversity of biofilm communities increased with the aging of MPs,^161,162^ and the relative abundance of some families of the microbial community is significantly altered after the aging treatment of MPs.^43,161,163^ Simultaneously, the genes associated with the biofilm formation were reported to be significantly expressed in photoaged MPs.^162^ In addition to providing novel substrates for biofilm formation, MPs can potentially facilitate the enrichment and spread of antibiotic-resistance genes (ARGs) and opportunistic human pathogens.^164,165^ Compared with pristine MPs, photoaged PS MPs enhanced selective ARG enrichment and ARG transfer due to increased proximal ARG donor-recipient adsorption and release of chemicals from MPs.^153,166^ Shan et al. suggested that photoaged PP MPs were more conducive to the expression of genes related to human pathogens,^161^ while the abundance of pathogen-related genes decreased with the aging of PE and PVC MPs.^162^ Different polymer types and photo-oxidation conditions may be the reason for the inconsistent results. Although these studies provide important information on the potential ecological and health risks of biofilm on aged MPs in aquatic ecosystems, detailed mechanisms underlying them remain to be determined.

#### 5.2.2 DOC Leaching and Planktonic Microbial Growth

MNPs are likely to contribute to the DOC pool in aquatic environments via leaching.^44^ Although plastic-fragments do leach DOC in the dark, light irradiation can stimulate the release of the plastic-derived DOC at levels more than 10 times higher than in the dark.^167−169^ The leached DOC from photo-oxidized MNPs shows low molecular weight and varies depending on polymer type.^45,167,170^ Plastic-derived DOC usually shows high lability and bioavailability.^45,169,170^ The bioavailability of the leached DOC depends on the plastic source and type.^169^ Among the postconsumer plastics, the bioavailability of ePS DOC (disposable lunch box) was the highest (76 ± 8%), followed by PP DOC (facial cleanser bottle) (59 ± 8%) and PE DOC (shampoo bottle) (46 ± 8%).^169^ Similarly, leached DOC from plastic shopping bags was chemically distinct and more bioavailable than NOM in lakes.^45^ However, Romera-Castillo et al. reported that the bioavailable fraction of the leached DOC under artificial solar radiation was (insignificantly) lower than that in the dark (55% ± 5% in the light treatments vs 61% ± 3% in the dark). They observed a lower bacterial abundance in the light treatments, which was explained by the possible generation of microbial inhibitors like ROS.^44^ In addition, harmful additives may be a source of the inhibitory effect of DOC.^169^ The harmful additives in the leachates from high-density PE (HDPE) bags and PVC matting were reported to strongly inhibit the growth of Prochlorococcus and photosynthetic capacity.^171^ Sheridan also suggested that high plastic leachate concentrations may further impair bacterial growth due to large quantities of toxic compounds (e.g., oxybenzone).^45^ Therefore, the plastic leachates after photo-oxidation can be biolabile or biotoxic, and how planktonic microbes respond to plastic leachates depends on their source and level, as well as the capacity of local microbial communities to utilize these leachates.

#### 5.2.3 Changing Microbial Communities in Soil and Sediment

Soil and sediment are major sinks of MNPs in aquatic and terrestrial ecosystems.^172^ As the primary life forms in soil and sediment systems, the microorganism is the main participator in many biogeochemical processes such as organic matter mineralization and nutrient cycling.^172,173^ The greatest attention has focused on the effect of MNPs on the microbial community and function in soil and sediment systems, including microbial growth and viability,^174^ microbial activity and enzyme activity,^175,176^ community structure, and function.^177−182^ Indirect mechanisms of these impacts come from changes in soil physicochemical parameters, including bulk density,^183^ porosity,^184^ soil aggregation,^185−187^ water-holding capacity,^188,189^ and pH.^173,186^ Direct interactions of MNPs with soil and sediment microorganisms are related to biofilm formation on MP surface,^175,190^ plastic leachate impact,^177^ ROS-induced oxidative stress,^191^ and combined impacts with other chemicals.^192,193^ The photo-oxidation of MNPs generally alters surface properties,^43^ leaching of toxic substances and DOC,^45,169^ and ROS production,^44^ and subsequently changes their stability,^27,31^ and mobility,^28,37^ adsorption,^69,71^ and transformations of contaminants.^56^ These effects of photoaged MNPs potentially lead to distinct alterations in microbial community and activity, although the specific response of soil characteristics and microorganisms to the photo-oxidation of MNPs remains largely unclear.^47,194^

For example, Liu et al. reported the photoaged tire wear particles (TWPs) were more toxic than pristine TWPs, which is attributed to the increased adsorption of released heavy metals due to the increase in specific surface area and the transition of positive to negative charge after photo-oxidation.^194^ The high levels of ROS produced by photoaged MNPs may induce oxidative stress in cells and subsequently suppress enzyme activity.^195−197^ Despite enzymes like superoxide dismutase, catalase, and peroxidase can scavenge ROS,^198^ research indicated that high ROS levels from UV-aged MPs may induce structural changes and denaturation in functional enzymes,^199^ suppressing soil microbial enzyme activity (e.g., fluorescein diacetate hydrolase).^47^ The polymer degradation byproducts from the aged MPs can be metabolized as substrates for specific microorganisms, which may result in a shift in microbial community composition.^47^ Aged PP and PS microfibers were shown to reduce the abundance of Sphingomonadales (oligotrophic bacteria) and increase the abundance of Burkholderiales (eutrophic bacteria) in soil compared with pristine MPs,^47^ potentially due to the restriction of the growth rate of oligotrophs by eutrophs.^200^ Furthermore, the photoaged MNPs may affect microbial metabolism processes such as carbon and nitrogen cycling. Chen et al. suggested that pristine PS MPs inhibited sediment bioavailability and CO_2_ emission, but this effect was decreased with the aging of MPs, which was explained by the utilization of DOC by sediment microbes as the carbon source to promote organic carbon mineralization.^46^ Rillig et al. speculated that aged MPs contain O-containing functional groups (e.g., ketones) that are redox active, which may serve as electron sinks and donors for microbes, and increase microbial metabolism efficiency.^201^ Although photoaged PE MPs did not significantly influence soil CO_2_ and N_2_O emissions compared with pristine MPs, aged MPs significantly increased soil NO_3_^–^ content and amoA gene abundance.^202^ It indicated that aged PE may increase the nitrification rate and then provide more substrates for denitrification, potentially increasing the emission of soil N_2_O.^202,203^ Although several studies provided some insights on the effects of photo-oxidation of MNPs on microbial community and function in soil and sediment, the processes and mechanisms are complicated and depend on various factors such as plastic properties, aging degree, and local microorganisms.

### 5.3 Potential Impacts on Plants

While the risks of MNPs to aquatic and terrestrial plants have been extensively documented,^204,205^ the potential impacts of photoaged MNPs on plants remain largely unexplored, and there is a dearth of information in this regard. The direct impacts of MNPs on plants include blockage of cell connections or pores in the cell wall,^206,207^ and the uptake, translocation, and accumulation in different plant parts such as roots, shoots, and leaves.^208,209^ The translocation of MNPs potentially posing toxic effects on various physiological and biochemical processes in plants, including inhibition of seed germination and plant development, biomass reduction, disruption of photosynthesis, oxidative damage, and genotoxicity.^206,210,211^ Surface properties (e.g., particle size, charge, and hydrophobicity) of MNPs play vital roles in the translocation of MNPs.^210^ Smaller sizes of MNPs increase the likelihood of entering seed pores and obstructing voids,^206^ and facilitating translocation in the vascular system and the cell walls of root tissues.^208,209^ Besides, the translocation of MNPs within the plant appears to favor negatively charged particles, likely attributed to electrostatic repulsions between MNPs and the electronegative cell walls.^212,213^ The translocation within plants could also be influenced by the aggregation states of MNPs.^214^ For instance, the growth medium and root exudates promoted the formation of large aggregates, restricting the uptake of positively charged PS NPs; conversely, negatively charged NPs tended to remain stable and can penetrate into root tissues.^214^ As discussed in sections 3.2 and 3.3, the typically more negative charges, hydrophilic nature, and smaller size contribute to the higher stability and mobility of photoaged MNPs in water and soil media compared to pristine ones.^27,28^ It can be expected that the photo-oxidation has the potential to increase the uptake and translocation of MNPs by plants, thereby inducing more adverse effects.

Apart from the physical blockage that can potentially inhibit water and nutrient adsorption,^206,207,215^ the physiological and biochemical responses of plants after MNPs exposure may arise from plastic leachates,^216^ and oxidative stress.^207,217^ For instance, Pflugmacher et al. highlighted the considerable toxicity of plastic leachates that induced a 77% decrease in the plant germination rate.^216^ Therefore, the photoaging can increase the leaching of toxic additives,^218,219^ potentially leading to adverse effects on plants. Besides, serving as scavengers or carriers of environmental pollutants,^69,73,80^ the photoaged MNPs may exert distinct impacts on plant growth in contaminated soils. The excessive ingestion of MNPs by plants can also lead to the production of ROS, causing irreversible damage to plant tissue and disrupting photosynthesis.^206,211,214^ Photoaged MNPs may induce more oxidative stress as they can produced more ROS or EPFRs,^220,221^ which exhibit specific toxicological properties, such as DNA damage, lipid peroxidation, protein oxidation, and inflammation.^220,222^ Although not previously reported, it is plausible that photoaged MNPs may result in more toxic effects through these chemical mechanisms.

Indirect impacts of MNPs on plants may also occur through alterations in soil physicochemical characteristics, such as pH,^186^ water holding capacity,^188,189^ and soil structure,^185−187^ as well as soil-dwelling microbes.^188,205^ Boots et al. indicated that the presence of HDPE MPs reduced the root growth of *Lolium perenne*, potentially attributed to the changes in soil pH and the size distribution of water-stable soil aggregates.^186^ Due to the formation of hydrophilic O-containing functional groups and DOC leaching, the photoaged MNPs potentially increase water and organic matter content, pH, and cation exchange ability in soils.^223,224^ These effects can indirectly impact plant growth such as seed germination and root growth.^188,225,226^ Furthermore, these changes in soil characteristics may impact soil fertility by influencing the growth of microbial communities in the rhizosphere—a crucial interface where plants interact with soil microorganisms.^226−228^ Recent findings suggested that polyester MPs enhanced microbial activity in both bulk soil and the rhizosphere.^188^ Additionally, the treatment with polyester promoted soil microbe colonization on spring onion roots, and the potential mycorrhizal symbiosis may facilitate the growth of plants subjected to polyester.^188^ The introduction of photo-oxidized MNPs into the soil adds another layer of complexity. They may exert distinct effects on soil microbial communities, which play a crucial role in plant-microbe interactions, and nutrient cycling and availability, potentially influencing plant health. In conclusion, our understanding of the potential risks of pristine and photoaged MNPs to plant ecosystems is currently limited, necessitating further comprehensive research.

## 6 Future Perspectives

### 6.1 Relate the Adsorption Capacity of MNPs to the Photo-Oxidation Degree and the Physicochemical Properties of Pollutants

Increasing studies have evaluated the effect of photo-oxidation of MNPs on their adsorption capacity toward contaminants, especially OPs. Generally, the photo-oxidation of MNPs may enhance the adsorption of hydrophobic OPs and reduce the adsorption of hydrophilic OPs due to enhanced O-containing functional groups.^77,79^ However, in some cases, it is complex and depends on many factors including polymer types of MNPs, oxidation degree, and physicochemical properties of OPs.^69,73,80^ There is a lack of knowledge to evaluate the effects of the photo-oxidation degree and the physicochemical properties of OPs on their adsorption processes on MNPs. Integrating experimental data with advanced analytical methods, such as molecular dynamic simulations^229^ and machine learning,^230,231^ offers a comprehensive approach for understanding the adsorption mechanism of MNPs after photo-oxidation, and establish the multidimensional relationship between the adsorption capacity of MNPs, the photo-oxidation degree (e.g., the carbonyl index and hydroxyl index, and the physicochemical properties of pollutants (e.g., molecular weight, log *K*_OW_, zeta potential, type, and number of functional groups).

### 6.2 Study the Combined Effect of Photo-Oxidation and Natural Colloids on the Fate of MNPs

The stability and mobility of MNPs in water and porous media are well reported to be influenced by the photo-oxidation process and coexisting natural colloids. However, in real-world environments, the effect of photo-oxidation and natural colloids can coexist. On the one hand, the photo-oxidation of MNPs may change their interaction with natural colloids (e.g., NOM, minerals and bacteria), and these colloids may have different effects on the stability and mobility of pristine and aged MNPs.^32,33,78^ On the other hand, MNPs are unlikely to undergo photo-oxidation alone, and the presence of natural colloids may take part in the photo-oxidation of MNPs and further affect the stability and mobility of MNPs in environments.^20,52,118^ More work is needed to better understand the combined effect of the photo-oxidation process and natural colloids on the fate of MNPs in aquatic and terrestrial environments. A well-structured experiment combining photo-oxidation with environmental transport and fate studies can be complemented by state-of-the-art particle detection techniques such as dynamic light scattering, nanoparticle tracking analysis, in situ microscopy, and Fourier Transform Infrared Spectroscopy (FTIR), as well as ROS detection techniques like electron paramagnetic resonance spectroscopy.

### 6.3 Link the Photochemical Transformation of Pollutants in the Presence of MNPs to the Physicochemical Properties of Pollutants

By generating ESPRs and ROS or light-screening effect, the coexisting MNPs may promote or inhibit the phototransformation of OPs. Although the promoting and inhibitory mechanisms have been studied for several OPs, the reasons why MNPs play different roles in the phototransformation of different types of OPs are unclear. In fact, the adsorption and phototransformation of OPs can occur simultaneously, and the chemical structure of OPs and the adsorption process may contribute to the distinct effect of MNPs on the photodegradation of OPs.^133^ Limited studies have considered the relationship between adsorption and photodegradation of OPs in the presence of MNPs. Thus, more research should associate the physicochemical characteristics of OPs such as hydrophobicity, electronegativity, and functional groups with their phototransformation process in the presence of MNPs, potentially utilizing molecular dynamic simulations,^232^ and machine learning.^233,234^

### 6.4 Pay Attention to the Effect of Photoaged MNPs on the Biotransformation of Pollutants

As reviewed, the photo-oxidation of MNPs significantly influences the physical adsorption and photochemical transformation of environmental pollutants. However, apart from the physicochemical process, microbial biotransformation of pollutants such OPs and HMs under aerobic or anaerobic conditions is also critical.^235,236^ Like the role of DOM in the biotransformation of pollutants,^237−239^ the photo-oxidation of MNPs is likely to affect the biotransformation of pollutants via several mechanisms: (1) the photo-oxidation may change the adsorption capacity of MNPs toward pollutants, and alter their bioavailability by microorganisms,^240^ (2) the high lability and bioavailability of DOC from oxidized MNPs may promote the cometabolic transformation of OPs,^45,169^ and (3) photoaged MPs or intermediates containing redox-active functional groups may serve as electron sinks and donors for microbes, and mediate the biotransformation of pollutants.^201,237^ So far, there has been no research on this topic, which should be paid more attention to in the future. There is potential for comprehensive investigations combining microcosm studies with advanced techniques such as metabolomics, proteomics, mass spectrometry, and electron paramagnetic resonance spectroscopy, to quantitatively assess the metabolism processes of OPs under the impact of photoaged MNPs.

### 6.5 Focus on the Interaction between Photoaged MNPs and Plants

The interaction between plants and MNPs is an emerging area of study with ecological importance. While the impacts of MNPs on plants have been explored to some extent, the effects of photo-oxidation on these impacts remain unclear. Techniques such as confocal microscopy and FTIR imaging spectroscopy hold the potential to detect the interactions between photoaged MNPs and plants, allowing for the visualization of spatial distribution and potential impacts.^241^ Additionally, advanced analytical methods like metagenomes and proteomics, coupled with plant physiology and biochemistry analyses and staining techniques, can be employed to probe the intricate biological responses of both plants and rhizosphere microbes when exposed to photoaged MNPs. Moreover, exploring plant interactions with pristine/weathered MNPs holds promise for addressing the adverse impacts of plastic contamination, contributing to the development of strategies for a cleaner and healthier planet.

## Supporting Information Available

Adsorption capacity and adsorption mechanism of photoaged MNPs toward OPs (Table S1); influence of photo-oxidation on the colloidal stability of NPs under different conditions (Table S2); critical factors influencing the photo-oxidation of MNPs (Table S3); effect of MNPs on the phototransformation of pollutants (Table S4); and biological effects of photo-oxidized MNPs on organisms and microbes (Table S5) (PDF)
