# Supplementary material for: Photo-oxidation of Micro- and Nanoplastics: Physical, Chemical, and Biological Effects in Environments
Source: Environ Sci Technol. 2024 Jan 3;58(2):991–1009. doi: 10.1021/acs.est.3c07035 (PMC10795193; doi:10.1021/acs.est.3c07035)
Supplement: Supplementary file 1 — es3c07035_si_001.pdf [file es3c07035_si_001.pdf]

Supporting Information

Photo-oxidation of Micro- and Nano-plastics: Physical, Chemical and Biological Effects in Environments

Yanghui Xu [a,b], Qin Ou [a,b], Jan Peter van der Hoek [b,c], Gang Liu [a,b] *, Kim Maren Lompe [b]

[a.] Key Laboratory of Drinking Water Science and Technology, Research Centre for Eco-Environmental Sciences, Chinese Academy of Sciences, Beijing, 100085, P. R. China

[b.] Section of Sanitary Engineering, Department of Water Management, Faculty of Civil Engineering and Geosciences, Delft University of Technology, Stevinweg 1, 2628 CN Delft, the Netherlands

[b.] Waternet, Department Research & Innovation, P.O. Box 94370, 1090 GJ Amsterdam, the Netherlands

Corresponding author:

Gang Liu,

Research Center for Eco-Environmental Sciences, Chinese Academy of Sciences

Sanitary Engineering, CiTG, Delft University of Technology

g.liu-1@tudelft.nl; gliu@rcees.ac.cn

The supplementary information includes 5 tables in 19 Pages.

Table S1. The adsorption capacity and adsorption mechanism of photo-aged MNPs towards OPs.

Contaminants	Log K_{OW}	Functional groups	MNPs	Radiation conditions	Adsorption mechanisms	Adsorption capacity	Reference
Benzene<br>Toluene<br>Ethyl benzene<br>*m*-Xylene<br>*o*-Xylene	2.13<br>2.73<br>3.15<br>3.20<br>3.12	Benzene (1)	PP,<br>PS	UV_{340};<br>6.27 kJ/cm^2;<br>4 weeks	π−π bond;<br>Hydrophobic interaction	Log K_D: Benzene: 1.01→0.68 (PS); 1.43→1.56 (PP);<br>Toluene: 1.37→1.14 (PS); 1.92→1.95 (PP);<br>Ethyl benzene: 1.60→1.40 (PS); 2.30→2.28 (PP);<br>*m*-Xylene: 1.57→1.32 (PS); 2.34→2.29 (PP);<br>*o*-Xylene: 1.26→1.02 (PS); 2.16→2.17 (PP)	1
Benzene	2.13	Benzene (1)	PS, PE,<br>PET,<br>PVC	UV/VUV;<br>180−1080 mJ/cm^2	π−π bond;	K_F (L/g): 0.653→0.612→0.459 (UV, PS); →0.361→0.105 (VUV, PS); 0.086→0.082→0.080 (UV, PE); →0.079→0.076 (VUV, PE); 0.267→0.215→0.168 (UV, PVC);→0.186→0.110 (VUV, PVC); 0.592→0.461→0.344 (UV, PET); →0.326→0.156 (VUV, PET);	2
Ciprofloxacin	1.81	Benzene (1)<br>Amino (1)<br>Carboxyl (1)			H bond;<br>π−π bond	K_F (L/g): 0.208→0.183→0.119 (UV, PS); →0.151→0.072 (VUV, PS); 0.141→0.121→0.112 (UV, PE); →0.116→0.108 (VUV, PE); 0.069→0.057→0.046 (UV, PVC);→0.071→0.088 (VUV, PVC); 0.219→0.181→0.111 (UV, PET); →0.192→0.171 (VUV, PET);	
Ciprofloxacin	1.81	Benzene (1)<br>Amino (1)<br>Carboxyl (1)	PS, PVC	UV_{254};<br>96 h	Electrostatic interaction;<br>H bond	K_F: 0.58→0.54 (PS); 0.55→0.74 (PVC);<br>K_L: 0.959→0.993 (PS); 0.966→0.969 (PVC);<br>K_d: 0.210→0.318 (PS); 0.215→0.251 (PVC)	3
Ciprofloxacin	1.81	Benzene (1)<br>Amino (1)<br>Carboxyl (1)	PVC,<br>PLA	UV_{313};<br>0.05−1.29 kJ/cm^2	Electrostatic interaction;<br>H bond	$Q_{e,1}$ (mg/g): 0.39→0.69 (PLA); 0.34→ 0.44 (PVC);<br>$Q_{e,2}$ (mg/g): 0.91→1.19 (PLA); 0.61→ 0.77 (PVC);<br>K_F (mg/g(L/mg)$^{1/n}$): 0.211→0.282 (PLA); 0.457→0.544 (PVC);<br>K_L (L/mg): 0.071→0.073 (PLA); 1.580→1.319 (PVC);<br>Q_{max} (mg/g): 3.19→3.77 (PLA); 0.67→0.85 (PVC);	4
Tetracycline	-0.07	Benzene (1)<br>Amino (1)<br>Hydroxyl (5)			Electrostatic interaction;<br>H bond	$Q_{e,1}$ (mg/g): 0.46→1.28 (PLA); 0.43→ 0.87 (PVC);<br>$Q_{e,2}$ (mg/g): 0.90→1.97 (PLA); 0.75→ 1.36 (PVC);<br>K_F (mg^{1-n}·L^n/g): 0.182→0.286 (PLA); 0.600→0.867 (PVC);<br>K_L (L/mg): 0.079→0.056 (PLA); 1.404→1.338 (PVC);<br>Q_{max} (mg/g): 2.51→5.49 (PLA); 0.96→1.57 (PVC);	
Pyrene	4.88	Fused Ring	PS	mercury lamp;<br>6−12 h	−	K_F (mg^{1-n}L^n/kg): 177000→279000→82700; n: 0.95→1.07→0.78	5
4-nonylphenol	4.28	Benzene (1)<br>Hydroxyl (1)			H bond	K_F: 19600→ 14500→ 31000; n: 1.03→0.93→0.78	
Sulfadiazine	-1.47	Benzene (1)<br>Amino (2)	TPU,<br>PA	mercury lamp;<br>96 h	H bond;	$Q_{e,1}$ (μmol/g): 0.676→0.499 (PA); 0.084→ 0.095 (TPU);<br>$Q_{e,2}$ (μmol/g): 0.727→0.536 (PA); 0.087→ 0.099 (TPU);<br>K_F (L/g): 0.025→0.030 (PA); 0.0021→ 0.0019 (TPU);	6

Contaminants	Log K_{OW}	Functional groups	MNPs	Radiation conditions	Adsorption mechanisms	Adsorption capacity	Reference
						n: 1.07→0.922 (PA); 1.11→1.18 (TPU);<br>K_d (L/g): 0.0309→0.0226 (PA); 0.0032→0.0036 (TPU);	
Sulfachlorpyridazine	0.90	Benzene (1)<br>Amino (2)			Electrostatic interaction	$Q_{e,1}$ (mg/g): 2.12→1.81 (PA); 0.293→ 0.324 (TPU);<br>$Q_{e,2}$ (mg/g): 2.24→1.92 (PA); 0.087→ 0.099 (TPU);<br>K_F (L/g): 0.688→0.256 (PA); 0.298→ 0.331 (TPU);<br>n: 0.858→0.907 (PA); 1.41→1.44 (TPU);<br>K_d (L/g): 0.529→0.201 (PA); 0.012→0.014 (TPU);	
Benzalkonium chlorides<br>BAC_{12} (C21H38NCl)<br>BAC_{14} (C23H42NCl)<br>BAC_{16} (C25H46NCl)	 2.93<br>3.91<br>4.89	Benzene (1)<br>Long-chain alkane	PE	UVC;<br>120 h	Hydrophobic interaction;<br>H bond	K_F (L/mg): 3.337→4.052 (BAC_{12}); 6.543→7.052 (BAC_{14}); 5.570→6.280 (BAC_{16});<br>1/n: 0.2177→0.2147 (BAC_{12}); 0.2064→0.1533 (BAC_{14}); 0.2696→0.2058 (BAC_{16});<br>K_L (L/mg): 0.0319→0.0373 (BAC_{12}); 0.0474→0.0744 (BAC_{14}); 0.0354→0.0526 (BAC_{16});<br>Q_{max} (mg/g): 12.6699→14.7535 (BAC_{12}); 22.0704→17.1392 (BAC_{14}); 26.9646→20.7013 (BAC_{16});	7
Atorvastatin	3.44	Benzene (3)<br>Carboxyl (1)<br>Hydroxyl (2)	PS	mercury lamp;<br>0.05−0.81 kJ/cm^2;	Hydrophobic interaction;<br>π−π bond;	$Q_{e,2}$ (mg/g): 1.24→0.59→1.33; K_d (L/g): 0.39→0.09→0.50;<br>$K_{d\text{-}SSA}$ (L/m^2): 1.21→0.08→0.24; $K_{d\text{-}O/C}$ (L/g): 52.91→2.07→4.25;<br>K_L (L/mg): 0.58→0.29; Q_{max}: 1.61→0.85→2.52;<br>K_F: 0.63→0.31→0.66; n: 0.28→0.28→0.40;	8<br>9
Amlodipine	2.08	Benzene (1)<br>Amino (2)			Electrostatic interaction;<br>H bond	$Q_{e,2}$ (mg/g): 0.22→1.10; K_d (L/g): 0.03→0.28;<br>$K_{d\text{-}SSA}$ (L/m^2): 0.08→0.05→0.14; $K_{d\text{-}O/C}$ (L/g): 3.40→1.18→2.39;<br>K_L (L/mg): 0.16→0.57; Q_{max}: 0.46→1.51;<br>K_F: 0.13→0.65; n: 0.31→0.25→0.25;	
Bisphenol A	3.03	Benzene (2)<br>Hydroxyl (2)	PS	UV_{365};<br>96 h	π−π bond;<br>Hydrophobic interaction	K_d (L/kg) : 18.8→1.4; $K_{d\text{-}CI}$ (L/kg): 348→6.0;<br>$Q_{e,1}$ (μg/g): 2.25→0.697; $Q_{e,2}$ (μg/g): 2.51→0.726	10
Atrazine	2.70	Amino (3)	PP,<br>PE,<br>PS	UV_{254};<br>96 h	Electrostatic attraction;<br>H bond	$Q_{e,1}$ (mg/g): 0.569→0.661 (PS); 0.519→0.935 (PE); 0.394→0.663 (PP); $Q_{e,2}$: 0.644→0.760 (PS); 0.598→1.104 (PE); 0.456→0.790 (PP);<br>K_L: 0.088→0.278 (PS); 0.038→0.227 (PE); 0.109→0.416 (PP); Q_{max} (mg/g):1.349→2.483 (PS); 1.992→3.539 (PE); 1.148→2.242 (PP);<br>K_F: 0.277→0.360 (PS); 0.249→0.446 (PE); 0.228→0.386 (PP); n: 0.429→0.399 (PS); 0.491→0.426 (PE); 0.424→0.369 (PP);	11
2,2`,4,4`-tetrabromodiphenyl ether	−	Benzene (2)	PS	UV_{254};<br>0.34 kJ/cm^2	π−π bond ;<br>Hydrophobic interaction	$Q_{e,2}$ (ng/L) : 6.105→3.755;<br>K_L (mL/ng): 0.107→0.136; Q_{max}: 50.761→7.342;<br>K_F (ng/g) (dm3/g): 11.92→5.33; n: 0.942→0.544	12

Contaminants	Log K_{OW}	Functional groups	MNPs	Radiation conditions	Adsorption mechanisms	Adsorption capacity	Reference
Sulfanilamide	-1.26	Benzene (1)<br>Amino (2)	PA,<br>PVC,<br>PET	–	Hydrophobic interaction;<br>Electrostatic interaction	$Q_{e,1}$ (μg/g): 17.178 (PVC); 13.977 (PA); 7.142 (PET);<br>$Q_{e,2}$ (μg/g) : 17.238 (PVC); 14.278 (PA); 7.249 (PET);<br>K_L (L/mg) : 0.05 (PVC); 0.039 (PA); 0.018 (PET);<br>Q_{max} (mg/g): 24.27(PVC); 19.70 (PA); 12.45 (PET);	[13]
Oxytetracycline	-0.90	Benzene (1)<br>Amino (1)<br>Hydroxyl (6)	TPU	UV_{254};<br>44.30 kJ/cm^2	H bond;<br>Cation bridging	$Q_{e,1}$ (mg/g): 0.280→0.372; 0.692→0.981 (with Cu^{2+});<br>$Q_{e,2}$ (mg/g): 0.318→0.398; 0.817→1.086 (with Cu^{2+});<br>K_L (L/mg): 0.137→0.070; 0.325→0.640 (with Cu^{2+});<br>Q_{max} (mg/g): 0.582→1.113; 0.980→1.506 (with Cu^{2+});<br>F_L ($mg^{1-n}\cdot L^n/g$): 0.141→0.137; 0.390→0.706 (with Cu^{2+});<br>n: 2.898→2.035; 4.062→4.319 (with Cu^{2+});	[14]

Note. $Q_{e,1}$ and $Q_{e,2}$ represent the amount of adsorption at equilibrium of the pseudo-first-order and pseudo-second-order model, respectively; Q_m and K_L are the theoretical maximum adsorption capacity and an equilibrium constant reflecting the adsorption energy of Langmuir model; K_F and n are the parameters reflecting adsorption capacity and the parameter reflecting adsorption strength of Freundlich model.

Table S2.The influence of photo-oxidation on the colloidal stability of NPs under different conditions.

Material information	Radiation condition	Surface properties	Critical results and CCC (mM) under different conditions	Reference
PS; 100 nm	UV_{365};<br>0.0006–0.147 kJ/cm^2;<br>1, 3, 5, 9, 12, 24 h	Size (nm): 120.1→80.2 (24 h);<br>ZP (mV): -43.03→-34.5→-44.1 (24 h);<br>WA (º); 108.2→ 90.7→73.7	NaCl (pH=6): 450→458→480→530→550→630→760;<br>$CaCl_2$: 33→27→21→18→16→12→8	[15]
PS; 100 nm	UV_{254} + H_2O_2;<br>60 and 120 h	CI: 0.05→0.41→0.75;<br>PI: 0.05→0.08→0.09	NaCl (pH=7.5): 591→957→1108	[16]
PS; 100 nm	mercury lamp;<br>12 and 24 h	WCA (º): 119.7→82.9→66.0;<br>PI: 0.02→0.05→0.17	NaCl (pH=5): 198→293→411; $CaCl_2$: 21.2→20.5→9.67;<br>NaCl (HA): 705→480→ND; $CaCl_2$ (HA): 12.3→14.1→ND;<br>NaCl (lysozyme): 169→73.9→ND; $CaCl_2$ (lysozyme): 10.2→12.7→ND;<br>NaCl (sodium alginate): 494→200→ND; $CaCl_2$ (sodium alginate): 23→17.7→ND	[17]
PS; 100 nm	UV_{254};<br>12 and 24 h	ZP (mV): -44.4→-41.4→-43.3;<br>Size (nm): 117.8→98.1→70.9;<br>PI: 0.07→0.11→0.18	NaCl (pH=7): 585→700→ND;<br>NaCl (HA): 1200→1100→ND;<br>NaCl (DBC): 500–550→635–680→ND	[18]
PS; 100 nm	UV_{365};<br>0.086–0.172 kJ/cm^2;<br>6, 12 and 24 h	ZP (mV): -40.1→-38.4;<br>Size (nm): 117.8→98.1→70.9;<br>PI: 0.16→0.18→0.21	NaCl (pH=6.5): 400→500→650→800; $CaCl_2$: 38→30→25→18;<br>NaCl (HA): ND; $CaCl_2$ (HA): 8→10→12→13;<br>UV reduced and enhanced NP stability with HA in NaCl and $CaCl_2$, respectively;<br>UV induced-flocculation of BSA molecules wrapped and destabilized NPs.	[19]
PS; 50, 200 and 1000 nm	mercury lamp;<br>10–120 min	no obvious change in the particle size distribution, and O/C	The effect of direct light irradiation on the aggregation of PS is negligible in PBS solution (1.0 mM, pH = 7.0).	[20]
PS, $PS-NH_2$, PS-COOH; 100 nm	UV_{365};<br>0.0007 $kJ/cm^2$0.5 h	ZP (mV): -59.9→-57.5 (PS); -41.5→-38.6 ($PS-NH_2$); -39.7→-40.6 (PS-COOH)<br>WCA (º): 65.4→66.7 (PS); 45.1→44.6 ($PS-NH_2$); 40.2→40.1 (PS-COOH)	UV irradiation reduced the stability of PS NPs in 200 mM NaCl, that of PS-COOH NPs in 20–50 mM $CaCl_2$, and that of $PS-NH_2$ NPs in 100–200 mM NaCl/Na_2SO_4 solutions.	[21]
PS, PS-COOH; 50 nm	No aging	Size (nm): 49 (PS); 47 (PS-COOH);<br>ZP (mV): -34 (PS); -40 (PS-COOH)	NaCl (pH=5): 264 (PS); 191 (PS-COOH); 478 (PS+BSA); 628 (PS-COOH+BSA);<br>$CaCl_2$: 29.1 (PS); 16 (PS-COOH); 42.2 (PS+BSA); 97.4 (PS-COOH+BSA);<br>Na_2SO_4: 200 (PS); 540 (PS-COOH); 267 (PS+BSA); 611 (PS-COOH+BSA);	[22]
PS, PS-COOH; 100 nm	No aging	Size (nm): 92.8 (PS); 97.3 (PS-COOH);<br>ZP (mV): -35.5 (PS); -34.7 (PS-COOH)	NaCl (pH=7.4): 310 (PS); 308 (PS-COOH); 410 (PS+1 mg/L HA); 393 (PS COOH+1 mg/L HA); 1138(PS+5 mg/L HA); 999 (PS-COOH+5 mg/L HA); $CaCl_2$: 28.9 (PS); 28.0 (PS-COOH); 31.8 (PS+1 mg/L HA); 39.7 (PS-COOH+1 mg/L HA); 27.0 (PS+5 mg/L HA); 27.0 (PS-COOH+5 mg/L HA);	[23]

Table S3. Critical factors influencing the photo-oxidation of MNPs.

Factors	MNPs	Radiation condition	Influence	Mechanism	Critical results	Reference
HA;<br>FA	PP	mercury lamp;<br>1.72–8.61 kJ/cm^2	inhibit	ROS scavengers and optical light filters	size change: 0.14 ± 0.03 vs 0.28 ± 0.02 (HA) vs 0.28 ± 0.01 (FA);<br>T_m (°C): 148.1 ± 0.47 vs 150.4 ± 0.53 (HA) vs 153.8 ± 3.06 (FA);<br>ZP (mV): −8.8 ± 0.64 vs −0.94 ± 2.13 (HA) vs −6.75 ± 3.73 (FA);	24
HA;<br>halide ions	PVC	UV_{365};<br>0.5–56 h	promote/ inhibit	produce $^3DOM^*$; sequester •OH	promote and inhibit organotin compounds release	25
HA;<br>FA	PS	mercury lamp;<br>4.30–34.4 kJ/cm^2	promote	produce •OH and $^1O_2$	CI: 0.026 (pristine), 0.15–0.18 (HA) vs 0.22 (FA)	26
FA	PS	mercury lamp;<br>10–120 min	promote	produce $^3FA^*$, •OH, and $^1O_2$	O/C: 0.03 (200 nm) vs 0.56 (200 nm, FA); 0.04 (1000 nm) vs 0.48 (1000 nm, FA)	20
pyrite	PS	xenon lamp;<br>3–30 d	promote	produce $O_2^{\bullet-}$, •OH and $^1O_2$	CI: 0.68 vs 0.86 (pyrite); PO/C 0.13 vs 0.40 (pyrite)	27
clay minerals	PVC,<br>PET	mercury lamp;<br>8.61–60.26 kJ/cm^2	promote	produce •OH	weight loss (%): 19.78 (PVC) vs 39.68 (PVC, kaolinite) vs 31.94 (PVC, montmorillonite); 20.22 (PET) vs 44.25 (PET, kaolinite) vs 36.99 (PET, montmorillonite)<br>O/C: 0.12 (PVC) vs 0.38 (PVC, kaolinite) vs 0.2 (PVC, montmorillonite); 0.15 (PET) vs 0.47 (PET, kaolinite) vs 0.24 (PET, montmorillonite);<br>CI: 0.15 (PVC) vs 0.33 (PVC, kaolinite) vs 0.26 (PVC, montmorillonite); 0.2 (PET) vs 0.35 (PET, kaolinite) vs 0.29 (PET, montmorillonite)	28
goethite;<br>hematite	PP,<br>PE	xenon lamp;<br>10.62–42.47 kJ/cm^2	promote	light-driven Fenton reaction and •OH	TOC (mg/L): 2.7 vs 4.2 (hematite) vs 7.2 (goethite);<br>CI: 0.49 (PP) vs 0.59 (PP, hematite) vs 0.64 (PP, goethite); 0.39 (PE) vs 0.49 (PE, hematite) vs 0.54 (PE, goethite);<br>weight loss (%): 36 (PP) vs 44(PP, hematite) vs 58 (PP, goethite); 27 (PE) vs 36 (PE, hematite) vs 48 (PE, goethite)<br>O/C: 0.33 (PP); 0.24 (PE)	29
soil components	PET	xenon lamp;<br>10.62–42.47 kJ/cm^2	promote/ inhibit	electrostatic interaction	weight loss (%): 11 vs 28 (MnO_2) vs 21 (SiO_2) vs 16 (Al_2O_3) vs 26 (goethite) vs 27 (hematite) vs 31 (kaolinite) vs 29 (montmorillonite) vs 5 (DOM) vs 5 (FA)	30
organic acids;	PVC	xenon lamp;<br>13.77 kJ/cm^2	promote	produce •OH	[Cl^-] (μM): 21.4 (2 μm) vs 24 (2 μm, oxalate) vs 31.6 (2 μm, oxalate-Fe(III)); 13.2 (10 μm) vs 17.2 (10 μm, oxalate) vs 23.8 (10 μm, oxalate-Fe(III)); 10.6 (25 μm) vs 16.4 (25	31

Factors	MNPs	Radiation condition	Influence	Mechanism	Critical results	Reference
Fe (III)					μm, oxalate) vs 22.2 (25 μm, oxalate-Fe(III)); 7.2 (150 μm) vs 9.9 (150 μm, oxalate) vs 12.4 (150 μm, oxalate-Fe(III));<br>SSA (m^2/g): 70.25 (2 μm) vs 74.27 (2 μm, oxalate) vs 81.87 (2 μm, oxalate-Fe(III)); 13.29 (10 μm) vs 13.91 (10 μm, oxalate) vs 15.35 (10 μm, oxalate-Fe(III)); 5.58 (25 μm) vs 5.7 (25 μm, oxalate) vs 6.36 (25 μm, oxalate-Fe(III)); 0.85 (150 μm) vs 0.88 (150 μm, oxalate) vs 0.97 (150 μm, oxalate-Fe(III));<br>WCA (°):102.9 (150 μm) vs 69.2 (150 μm, oxalate) vs 35.4 (150 μm, oxalate-Fe(III)) vs 99.7 (150 μm, citrate) vs 52 (150 μm, citrate-Fe(III))	
brominated flame retardants	PS	xenon lamp; 0.13–353.21 kJ/cm^2	promote	produce Br• radicals	M_w (KDa): 0.18*10^5 vs 0.012*10^5 (BDE-209);<br>BFRs enhanced photo-oxidation rate of PS by 7 for TBBPA-DBPE and TBBPA, and 10 for BDE-209.	[32]
PS and its released DOM	PS, PP	mercury lamp; 3–25 d	promote	produce •OH and $^1O_2$	size distribution (%): 76.9 (> 100 μm) vs 62.5 (> 100 μm, virgin PS) and 66.2 (> 100 μm, aged PS);<br>0 (< 10 μm) vs 85.2 (< 10 μm, virgin PS) and 96.3 (< 10 μm, aged PS); 1.98 (< 1 μm) vs 24.5 (< 1 μm, virgin PS) and 20.6 (< 1 μm, aged PS);<br>CI: 0.32 vs 0.41 (PS-DOM);<br>average size (μm): 3 vs 1.5 (PS-DOM)	[33]
iron red pigment	PP	xenon lamp; 1.8 mW/cm^2; 10–120 d	inhibit	light shielding and compete for electrons and ROS	T_m (°C): 132.8 vs 147.2 (Fe_2O_3);<br>X_c: 0.48 vs 0.71 (Fe_2O_3);<br>WCA (°):108.3→91.7 vs 102.8→92.3 (Fe_2O_3);<br>O/C: 0.009→0.081 vs 0.012→0.079 ((Fe_2O_3);	[34]
antioxidant	PP	mercury lamp; 1.55–9.30 kJ/cm^2	inhibit	sequester •OH	CI: 0.12→0.5 (pure PP) vs 0.01→0.16 (meal box PP) vs 0.05→0.19 (tea cup PP);<br>O/C: 0.02→0.14 (pure PP) vs 0.02→0.05 (meal box) vs 0.02→0.05 (tea cup);<br>X_c: 0.62→0.564 (pure PP) vs 0.613→0.612 (meal box) vs 0.612→0.607 (tea cup);<br>T_m (°C): 166→159 (pure PP) vs 156→149 (meal box) vs 167→149 (tea cup);<br>ZP (mV): -1.37→-6.12 (pure PP) vs 2.5→2.5 (meal box) vs 0→-2 (tea cup);<br>size (μm): 444→34 (pure PP) vs 443→60 (meal box) vs 443→200 (tea cup)	[35]
NO_3^-, Br^-, Cl^-, HCO_3^-,	PS	xenon lamp; 5–40 d	promote/ inhibit	NO_3^-, Br^- and Cl^- produce ROS; HCO_3^- sequester •OH	size (μm): 27 (UP) vs 26 (HCO_3^-) vs 20 (NO_3^-) vs 23 (Cl^-) vs 22 (Br^-);<br>O/C: 0.203 (UP) vs 0.215 (HCO_3^-) vs 0.246 (NO_3^-) vs 0.221 (Cl^-) vs 0.242 (Br^-);<br>M_w (KDa): 229.8 (UP) vs 230.9 (HCO_3^-) vs 179.5 (NO_3^-) vs 188.1 (Cl^-) vs 184.7 (Br^-);<br>M_n (KDa): 77.1 (UP) vs 76.0 (HCO_3^-) vs 45.1 (NO_3^-) vs 51.4 (Cl^-) vs 48.0 (Br^-);<br>M_p (KDa): 151.4 (UP) vs 149.8 (HCO_3^-) vs 127.1 (NO_3^-) vs 133.0 (Cl^-) vs 120.9 (Br^-);	[36]

Factors	MNPs	Radiation condition	Influence	Mechanism	Critical results	Reference
Cl^-	PP	mercury lamp; 1.72–17.22 kJ/cm^2	inhibit	formation of $Cl_2•^-$ in seawater could react with $HO_2•$ and prevent the formation $O_2•^-$	PDI: 2.98 (UP) vs 3.04 (HCO_3^-) vs 3.98 (NO_3^-) vs 3.66 (Cl^-) vs 3.85 (Br^-); CI: 1.609 (UP) vs 1.643 (HCO_3^-) vs 1.762 (NO_3^-) vs 1.671 (Cl^-) vs 1.706 (Br^-); O content (%):0.50 ± 0.14 (pristine); 2.95 ± 0.63 (UP) vs 1.35 ± 0.07 (estuary) vs 1.30 ± 0.28 (seawater); ZP (mV):1.94 ± 0.64 (pristine); -10.4 ± 4.71 (UP) vs -7.37 ± 1.76 (estuary) vs -7.07 ± 1.18 (seawater); WCA (°):103 (pristine); 89.8 (UP) vs 97.9 (estuary) vs 98.2 (seawater); T_m (°C): 166.2 (pristine); 147.3 (UP) vs 147.4 (estuary) vs 156.3 (seawater); X_c (%):98.56 ± 0.98 (pristine); 91.75 ± 0.46 (UP) vs 95.53 ± 0.65 (estuary) vs 98.16 ± 4.15 (seawater)	[37]
Cu^{2+}, Pb^{2+}	PS	xenon lamp; 1–7 d	promote	produce ROS	CI: 0.03 (pristine); 0.034 (UP) vs 0.048 (Cu^{2+}) vs 0.086 (Pb^{2+}); O/C: 0.013 (pristine); 0.022 (UP) vs 0.029 (Cu^{2+}) vs 0.035 (Pb^{2+});	[38]
nano-ZnO	PS	xenon lamp; 4.73–18.94 kJ/cm^2	promote	produce •OH, $^1O_2$	PS MPs were almost photodegraded with ZnO NPs after 96 h irradiation.	[39]

Notes. T_m: melting temperature; ZP: zeta potential; CI: carboxyl index; O/C: ratio of O content to C content; X_c: the degree of crystallinity; WCA: water contact angle. SSA: specific surface area; M_w: weight-average molecular weight; M_n: number-average molecular weight; M_p: peak molecular weight; PDI: polydispersity index. μm: micrometer (particle size unit of MNPs). DBE-209: decabromodiphenylether; TBBPA: tetrabromobisphenol A; TBBPA-DBPE: tetrabromobisphenol A-bis (2.3-dibromopropylether).

Table S4. The effect of MNPs on the photo-transformation of pollutants.

Contaminants	MNPs	radiation condition	Influence	Mechanism	Critical results	Reference
Sulfamethoxazole	pristine and aged PS, 100 mg/L	mercury lamp; 68.5 mW/cm^2; 2 h	inhibit oxidation	light screening	k_{obs} (min^{-1}): 0.0025 vs 0.0024 (PS_0) vs 0.0023 (PS_{36}) vs 0.0017 (PS_{72}) vs 0.0014 (PS_{120}); degradation rate (%): 25.9 vs 24 (PS_0) vs 24 (PS_{36}) vs 18.3 (PS_{72}) vs 15.6 (PS_{120}); $t_{1/2}$ (min): 277 vs 289 (PS_0) vs 301 (PS_{36}) vs 408 (PS_{72}) vs 495 (PS_{120});	40
Sulfamethoxazole	PS- and PE-DOM; 5–30 mg/L	xenon lamp; 2 h	inhibit oxidation	light screening	degradation rate (%): 28.1 vs 19.5 (5 mg/L PS-DOM) vs 17.7 (10 mg/L PS-DOM) vs 14 (20 mg/L PS-DOM) vs 8.16 (30 mg/L PS-DOM) vs 33.4 (10 mg/L SRNOM); k_{obs} (10^{-5} s^{-1}): 3.75 vs 2.48 (10 mg/L PS-DOM) vs 4.38 (10 mg/L SRNOM)	41
Pyrene	pristine PS; 1–20 mg/L	xenon lamp and UV_{315} light; 3 h	inhibit oxidation	light screening; quenching	k_{obs} (10^{-2} min^{-1}): 0.54 vs 0.47 (1 mg/L 0.1 μm PS) vs 0.43 (20 mg/L 0.1 μm PS) vs 0.35 (1 mg/L 1 μm PS) vs 0.35 (20 mg/L 1 μm PS);	42
Anthracene			promote oxidation	$^1O_2$, $O_2{\bullet}^-$ and •OH	k_{obs} (10^{-2} min^{-1}): 0.82 vs 1.03 (1 mg/L 0.1 μm PS) vs 1.11 (20 mg/L 0.1 μm PS) vs 0.94 (1 mg/L 1 μm PS) vs 1.01 (20 mg/L 1 μm PS)	
Tetrabromodiphenyl ether	aged PS, PE and PP	xenon lamp; 860 mW/cm^2; 24 h	inhibit/ promote oxidation	light shielding	degradation rate (%): 96.2 (PS_0) vs 50 (PS_{48}); 90.1 (PP_0) vs 96.8 (PP_{48}); k_{obs} (min^{-1}): 0.095 (PP_0) vs 0.148 (PP_{48}); 0.123 (PE_0) vs 0.174 (PE_{48})	43
Cimetidine	pristine and aged PS; 0.4 g/L	xenon lamp; 70 mW/cm^2; 2 h	promote oxidation	$^1O_2$ and $^3PS^*$	degradation rate (%): < 8 (PS_0) vs 93 (PS_{3d}); > 99 (PS_{5d}) vs 99 (PS_{7d}); k_{obs} (h^{-1}): 0 (PS_0) vs 1.16 (PS_{3d}); 3.37 (PS_{5d}) vs 2.27 (PS_{7d})	44
Atorvastatin	pristine and aged PS; 0–0.5 g/L	xenon lamp; 1.65 mW/cm^2; 3 h	promote oxidation	$^1O_2$ and $^3PS^*$	k_{obs} (h^{-1}): 0.090 vs 0.100 (0.001 g/L) vs 0.103 (0.01 g/L) vs 0.170 (0.1 g/L) vs 0.250 (0.5 g/L); $t_{1/2}$ (h): 7.69 vs 6.90 (0.001 g/L) vs 6.71 (0.01 g/L) vs 4.07 (0.1 g/L) vs 2.78 (0.5 g/L); degradation rate (%): 19.82 vs 21.02 (0.001 g/L) vs 22.57 (0.01 g/L) vs 32.86 (0.1 g/L) vs 50.27 (0.5 g/L); k_{obs}= 0.285CI + 0.158 (0.1 g/L; R^2=0.933)	45
Cefazolin	pristine and aged PVC; 1g/L	mercury lamp; 7 d	promote oxidation	H bonds	k_{obs} (10^{-3} h^{-1}): 1.1±0.09 vs 1.0±0.02 (pristine PVC) vs 3.4±0.04 (aged PVC);	46
Cephalexin			no effect		k_{obs} (10^{-3} h^{-1}): 1.5±0.05 vs 1.3±0.06 (pristine PVC) vs 1.4±0.08 (aged PVC);	
Tetracycline	PS; 1.25–6.25	xenon lamp;	promote	$^1O_2$, $O_2{\bullet}^-$	k_{obs} (h^{-1}): 0.066 vs 0.078 (PS);	47

Contaminants	MNPs	radiation condition	Influence	Mechanism	Critical results	Reference
	g/L	5 h	oxidation	and •OH	$t_{1/2}$ (h): 10.45 vs 8.92 (PS); degradation rate (%): 27.65 vs 39.47 (PS)	
Organotin	PP, PE, PS, PMMA; 1 g/L	UV_{365} lamp; 2.3 mW/cm^2; 8 h	inhibit oxidation	light screening	degradation rate: control (without MPs) > PMMA > PS > PE > PP	48
PP MPs	pristine and aged PS; 0.4–1 g/L	UV lamp; 0–25 d	promote oxidation	•OH	ln(d/d_0) (d^{-1}): 0.22 (PP) vs 0.25 (PP + pristine PS) vs 0.26 (PP + aged PS); size percent (<1 µm) (%): 1.98 (PP) vs 24.5 (PP + pristine PS) vs 20.6 (PP + aged PS); CI: 0.32 (PP) vs 0.35 (PP + 0.4 g/L pristine PS) vs 0.41 (PP + 1 g/L pristine PS) vs 0.41 (PP + 0.4 g/L aged PS) vs 0.43 (PP + 1 g/L aged PS) vs 0.41 (PP + 50 mg/L PS-DOM)	33
PS MPs	aged BMP-DOM;	UV lamp; 0–5 d	promote oxidation	$^1O_2$ and O_2•$^-$	size (µm): 206 ± 24 (pristine); 175 ± 46 vs 116 ± 25 (PBOM) vs 131 ± 25 (PLOM); size percent (100–180 µm) (%): < 10 (pristine); 52.9 vs 94.1 (PBOM) vs 86.7 (PLOM); O/C: 0.034 (pristine); 0.112 vs 0.176 (PBOM) vs 0.125 (PLOM);	49
Cr(VI)	pristine and aged PS; 10 mg/L	xenon lamp; 55 mW/cm^2; 90 h	promote/ inhibit reduction	O_2•$^-$	Cr(VI) removal (%): 0.65 (dark, PS) vs 23.78 (light, PS); Cr(VI) reduction rate (h^{-1}): 0.0029 (PS-0) vs 0.0023 (PS-200) vs 0.0019 (PS-500) vs 0.0017 (PS-800)	50
Cr(III)	PS, PA, PVC, PP	UV_{254}; 1 mW/cm^2; 120 h	inhibit oxidation	compete ROS	photo-oxidation rate: PS > PA > PVC > PP > control	51
Ag^+	pristine and aged PS; 20 mg/L	xenon lamp; 55 mW/cm^2; 24 h	promote reduction	$^1O_2$ and •OH	[Ag^+] (mg/L): 8.5⟶7.35(dark) vs 7.93 (dark, PS) vs 7.19 (light) vs 6.73 (light, PS); Ag^+ reduction rate (h^{-1}): 0.054 (pristine PS) vs 0.117 (aged PS) vs 0.086 (PS-NH_2) vs 0.048 (PS-COOH)	52
Ag^+	PS; 500 mg/L	xenon lamp; 50 mW/cm^2; 24 h	promote reduction	electron shuttling	[Ag^+] (mg/L): 48.3 ± 1.8 (light, PS)vs 43.9 ± 2.7 (light, PS)	53
Mn^{2+}	PS; 10 mg/L	xenon lamp; 6 h	promote oxidation	ROO• and O_2•$^-$	[MnO_2] (µM): 3 (30 nm PS-COOH, without buffer) vs 47 (30 nm PS-COOH) vs 6 (100 nm PS-bare) vs 14 (100 nm PS-COOH) vs 17 (100 nm PS-NH_2)	54
Nano-Ag	PS; 20 mg/L	xenon lamp; 55 mW/cm^2; 96 h	promote oxidation	$^1O_2$, •OH and acid release	[Ag^+] (mg/L): 0.64 vs 0.97 (PS)	55
Nano-ZnO	PS; 20 mg/L	xenon lamp; 55 mW/cm^2; 96 h	promote dissolution	$^1O_2$, •OH and acid release	[Zn^{2+}] (mg/L): 3 vs 11 (PS)	39

Table S5. The biological effects of photo-oxidized MNPs on organisms and microbes.

Objects	Dose condition	Properties of MNPs (pristine vs aged)	Effect	Description	Reference
Zebrafish	PA (5, 20, and 50 mg/L)	WCA (°): 90.6 vs 83.3 (UV) vs 57.3 (UV, H_2O_2);<br>O/C: 0.43 vs 0.6 (UV, H_2O_2);<br>N/C: 0.27 vs 0.23 (UV, H_2O_2);<br>EPFRs: $-CH_2$•, −CH•, O=C• (UV, H_2O_2);<br>size (μm): 8.13 vs 6.73 (UV, H_2O_2);<br>size after 12 h (μm): 17.2 vs 16.3 (UV) vs 10.2 (UV, H_2O_2);	reduced toxicity	malformation rate (%): 3.6 (blank) vs 11.5 vs 5.1 (UV, H_2O_2);<br>ROS level (%): 204.3 vs 162.7 (UV) vs 119.3 (UV, H_2O_2);<br>tumor necrosis factor α level (%): 61.6 vs 10.9 (UV, H_2O_2);<br>interleukin 6 level (%): 62.3 vs 16.2 (UV, H_2O_2);<br>body burden (mg/g): 1.28 vs 1.24 (UV) vs 0.92 (UV, H_2O_2);<br>gene number upregulated: 131 vs 69 (UV, H_2O_2);<br>gene number downregulated: 244 vs 126 (UV, H_2O_2);	[56]
Zebrafish	PA (100 μg/L); tris(1,3-dichloro-2-propyl) phosphate (TDCIPP) (0.4, 2, and 10 μg/L)	SSA (m^2/g): 3.8 vs 1.4;<br>O/C: 0.43 vs 0.6; N/C: 0.27 vs 0.23;<br>WCA (°): 94.8 vs 52.6;<br>size (μm): 17.4±7.2 vs 9.2±3.7;<br>TDCIPP Q_e (mg/g): 0.363 vs 0.789	reduced toxicity	[TDCIPP] (μg/kg) in adult zebrafish: 74.3, 223.1, and 735.9 vs 99.3, 359.3, and 1053.3 (pristine PA) vs 81.9, 269.3, and 832.5 (aged PA); F0 zebrafish intestine: aged PA + TDCIPP > pristine PA + TDCIPP > TDCIPP; F0 zebrafish gonad: aged PA + TDCIPP < pristine PA + TDCIPP; F1 larvae: pristine PA + TDCPP > > TDCIPP > aged PA + TDCIPP;<br>[MPs] (particle/g) in adult zebrafish: 243.3−266.5 vs 239.5−251.5; F0 gonad: 3.51−3.71 vs 3.09−3.55; F1 larvae: 0 vs 0	[57]
Zebrafish	PLA;<br>0.1−25 mg/L	size (μm): 25.56 vs 11.22;<br>WCA (°): 74.2 vs 47.1;<br>ZP (mV): -16.6 vs -27.9;<br>EPFRs: C-centred	enhanced toxicity	body length (mm): 4.5 vs 4.1;<br>cellular content (mg/g): 0.28 and 0.56 vs 0.4 and 0.78;<br>caspase activity (%): 114 and 153 vs 176 and 208;<br>gene number upregulated: 192 vs 242;<br>gene number downregulated: 88 vs 170;	[58]
Zebrafish	PA; 32.50 μm;<br>1, 10, and 20 mg/L;<br>HA and FA	size (μm): 32.5 vs 24.3 (HA) vs 19.8 (FA);<br>O/C: 0.45 vs 0.46–0.50 (HA) vs 0.51–0.63 (FA);<br>EPFRs: O-centred (HA, FA);<br>WCA (°): 80.6 vs 58.8 vs 52.1;<br>ZP (mV): -10 vs -25 (HA) vs -28 (FA);	enhanced toxicity	hatching rate (%): 90.7 (blank) vs 88.9 vs 84.1 (HA) vs 79.7 (FA);<br>body weight decrease (%): 12.8 vs 22.2 (HA) vs 29.8 (FA);<br>ROS level (%): 146.7 vs 191.3 (HA) vs 268.4 (FA);<br>MP content (mg/g): 0.66 and 0.95 vs 0.81 and 1.17 (HA) vs 0.93 and 1.37 (FA);	[59]
Carassius auratus	PS (100 μg/L); roxithromycin (ROX)	O-containing groups occurred	enhanced toxicity	ROX in intestine (ng/g): 460 (ROX) vs 660 (ROX, pristine PS) vs 770 (ROX, aged PS)	[60]

Objects	Dose condition	Properties of MNPs (pristine vs aged)	Effect	Description	Reference
Grouper	PS (0.01−130 mg/g)	size (μm): 55.9±25.5 vs 38.6±24.6;<br>O/C: 0.04 vs 0.22;<br>O-containing groups (%): 42.8 vs 52.1;<br>EPFRs: O-centred (aged);	enhanced toxicity	MP content (mg/g): 6.01±0.17 vs 8.21±0.30 ;<br>Additives in liver (μg/kg): 0 (pristine) vs dibutyl phthalate (9.60±0.47), di-isobutyl phthalate (1.54±0.09), acetophenone (0.54±0.05), and benzaldehyde (0.37±0.03) (aged).<br>growth inhibition extent: aged > pristine;<br>hepatomegaly extent: aged > pristine	[61]
Tilapia	PS (10 μg/L); sulfamethoxazole (SMX) and propranolol (PRP) (50 μg/L)	size (μm): 5.1±0.36 vs 5.0±0.35;<br>SSA (m^2/g): 5.47 v 6.92;<br>average pore diameter (nm): 4.56 vs 4.13;<br>SMX Q_e (μg/g): 1882 vs 2024;<br>PRP Q_e (μg/g): 1885 vs 1928;	enhanced/ reduced toxicity	[PRP] (μg/kg) in liver: 2500 (control) vs 4750 vs 3500; gut: 590 vs 16500 vs 7500; brain: 1400 vs 18000 vs 33000; gill: 1700 vs 3300 vs 2200;<br>[SMX] (μg/kg) in gut: 450 vs 500 vs 480; gill: 48 vs 138 vs 78	[62]
Daphnia magna	PE (14−71 mg/L); benzalkonium chlorides (BAC) (0.5 mg/L)	SSA (m^2/g): 0.06 vs 0.78;<br>ZP (mV): -11.7 vs -53.3;<br>CI: 0.035 vs 0.044;<br>BAC_{12} Q_e (mg/g): 12.7 vs 14.8;<br>BAC_{14} Q_e (mg/g): 22.1 vs 17.1;<br>BAC_{16} Q_e (mg/g): 27.0 vs 20.7;	enhanced/ reduced toxicity	survival rate (%): 30 (BAC_{12}) vs 74 (BAC_{12}, PE) vs 100 (BAC_{12}, aged PE); 0 (BAC_{16}) vs 64 (BAC_{16}, PE) vs 50 (BAC_{16}, aged PE)	[7]
Daphnia magna	PS (1 mg/L); nano-ZnO (5 mg/L)	[Zn^{2+}] (mg/L): 0.17 (ZnO, dark) vs 0.17 (ZnO, PS, dark) vs 0.76 (ZnO, UV) vs 2.93 (ZnO, PS, UV) ;	enhanced toxicity	mortality (%): 20 (ZnO, dark) vs 16 (ZnO, PS, dark) vs 50 (ZnO, UV) vs 88.5 (ZnO, PS, UV) ;<br>proporaion of ion-related toxicity (%): 2.68 (ZnO, dark) vs 36.15 (ZnO, UV) vs 92 (ZnO, PS, UV)	[39]
Daphnia magna	PS (1 mg/L); nano-Ag (50 μg/L)	[Ag^+] (mg/L): 0.41 (Ag, dark) vs 0.41 (Ag, PS, dark) vs 1.3 (Ag, UV) vs 2.7 (Ag, PS, UV) ;	enhanced toxicity	mortality (%): 8.5 (Ag, dark) vs 10.4 (Ag, PS, dark) vs 49.6 (Ag, UV) vs 95.2 (Ag, PS, UV) ;<br>proporaion of ion-related toxicity (%): 1.58 (Ag, dark) vs 29.11 (Ag, UV) vs 42.5 (Ag, PS, UV)	[55]
Chlorella vulgaris	PS and PVC (10−1000 mg/L); Cu^{2+} and Cd^{2+}	ZP (mV): -30 vs -26 (PS); -22 vs -19 (PVC)	enhanced/ reduced toxicity	growth inhibition rate (%): 11.46−29.1 vs 17.65−36.84 (PS); 5.27−14.55 vs 11.15−30.03 (PVC);<br>growth inhibition rate (%): 25.7 (aged PS) vs 17.34 (aged PVC) vs 19.5 (Cu^{2+}) vs 85.14 (Cd^{2+}) vs 10.22 (aged PS, Cu^{2+}) vs 27.55 (aged PS, Cd^{2+}) vs 18.89 (aged PVC, Cu^{2+}) vs 32.51 (aged PVC, Cu^{2+})	[63]

Objects	Dose condition	Properties of MNPs (pristine vs aged)	Effect	Description	Reference
Chlamydomonas reinhardtii	PVC (10−200 mg/L)	SSA (m^2/g): 0.49 vs 1.02; ZP (mV): -30.3 vs -12.7	enhanced toxicity	EC_{50} (mg/L): 104.93 vs 63.66; chlorophyll a content (μg/L): 3152 (control) vs 2667 vs 2571	64
Caenorhabditis elegans	PS (0.1–100 μg/L)	size (μm): 1.005±0.009 vs 1.014±0.130; CI: 0.085 vs 0.442; O/C: 0.031 vs 0.147	enhanced toxicity	head thrashes (%): 83.2 vs 68.8; body bends (%): 80.08 vs 76.0	48
Caenorhabditis elegans	PS (0.1–100 μg/L)	size (μm): 1.010±0.037 vs 0.996±0.012; CI: 0.14 vs 0.388; O/C: 0.062 vs 0.157	enhanced toxicity	brood size (%): 76.5 vs 68; number of egg ejection (%): 83.4 vs 77.7; number of foci (%): 100 (control) vs 217 (aged PS)	65
Scenedesmus obliquus	PS (1 mg/L); EPS	ZP (mV): -17.0 (PS) vs -14.9 (PS, EPS) vs -14.3 (PS, UV) vs -13.2 (PS, EPS, UV); size (nm): 238 (PS) vs 238 (PS, EPS) vs 925 (PS, UV) vs 1071 (PS, EPS, UV);	reduced toxicity	cell viability (%): 68.6 (PS, EPS) vs 91.5 (PS, PES, UV); superoxide dismutase activity (%): 153.8 (PS, EPS) vs 120.5 (PS, PES, UV); catalase activity (%): 648.6 (PS, EPS) vs 162.2 (PS, PES, UV); cell permeability (%): 123.3 (PS, EPS) vs 216.7 (PS, PES, UV);	66
Fungi	PE, PP, PET; methylene blue	TOC (mg/L): 4 vs 17 (PE); 2.3 vs 15 (PVC); 0.2 vs 17 (PP)	−	decolourization rate (%): 5.75 (control) vs 28.56 (PE leachate); 53.57 (control) vs 50.9 (PP leachate) vs 40.6 (PET leachate)	67
Bacteria	mSBR (100–1000 mg/L)	size (μm): 3.074 vs 2.297; WCA (°): 147.6→151.3→145; ZP (mV): -25.1→-27.2→-32.2→-33.1	enhanced toxicity	OD600 (A/A_0): 0.93 vs 0.88; protein (A/A_0): 0.71 vs 0.63	68
Biofilm	PBAT and PVC (1.2–1.7 items/mL)	MP generation (items/g): 428.67±300.46 −1473.27±143.67 (PBAT); 185.53±85.73−584±506.12 (PVC)	enhanced growth	OUT number: 104 vs 190−314 (PBAT); 54 vs 172−214 (PVC); Shannon index: 1.15 vs 3.22−3.59 (PBAT); 0.44 vs 3.13−3.62 (PVC); Chao 1 index: 111 vs 240−319 (PBAT); 63 vs 214−341 (PVC)	69
Biofilm	PE (5 g/L)	WCA (°): 122 vs 98 (5 mm); formation of carbonyl and vinyl groups	enhanced growth	protein content (μg/g): 498 vs 917 (1 mm); 895 vs 1504 (5 mm); OUT number: 391 vs 295 (1 mm); 371 vs 213 (5 mm); Shannon index: 5.1 vs 3.6 (1 mm); 5.8 vs 2.5 (5 mm); Chao 1 index: 395 vs 296 (1 mm); 273 vs 219 (5 mm)	70
Biofilm	PE	surface generated pores and became rough; O/C: 0.119−0.129 vs 0.154−0.183	–	OD595: 0.073–0.281 vs 0.263–0.414 (2 weeks); OUT number: 237–306 vs 251–907; Shannon index: 7.07 vs 7.59; Chao 1 index: 1946 vs 2161	71

Objects	Dose condition	Properties of MNPs (pristine vs aged)	Effect	Description	Reference
Sediment microorganisms	PS (0.5 %)	CI: 0.028 vs 0.069;	reduced negative effect	DOM humification index: 0.39–2.31 (control) vs 0.60–2.83 vs 0.42–2.52;<br>DOM biological index: 0.73–1.21 (control) vs 0.69–0.98 vs 0.60–0.99;<br>DOM bioavailability: 0.92 (control) vs 0.78 vs 0.71	72
Sediment microorganisms	TW (0.3 g/L)	SSA (m^2/g): 0.098 vs 0.139;<br>ZP (mV): 1.78 vs -0.72	enhanced toxicity	B. subtilis viability (%): 35 vs 32 (12 h); 73 vs 64 (48 h);<br>H. lutimaris viability (%): 89 vs 81 (12 h); 48 vs 35 (48h)	73
Soil microorganisms	PE (1 %)	CI: 0.28 vs 0.42	–	pristine and aged MPs increased total CO_2 emissions by15.8–36.1 and 10.0–14.4 for two types of soils;<br>pristine and aged MPs increased total N_2O emissions by 13.6–41.9, 3.5–9.5 and 8.9–16.8 folds for three types of soils.	74
Soil microorganisms	PE (0.01–1 %)	–	–	microbial biomass carbon (MBC) (mg C/kg): 213.3 (control) vs 206.7–210.9 vs 217.2–238.3;<br>metabolic quotient (mg CO_2-C/mg MBC): 0.59–0.72 vs 0.54–0.64;<br>soil DOC (mg C/kg): 50.8–55.9 vs 55.5–65.8;<br>soil NO_3^- (mg N/kg): 11.3–14.2 vs 12.4–14.6	75
Soil microorganisms	PP and PS (5 %)	formation of O-containing groups	enhanced negative effect	enzyme activity (μg-fluorescein/g-soil·h): 73.8 (control) vs 59.2 vs 49 (PP); 73.8 (control) vs 61.0 vs 57.8 (PS);	76
Planktonic microorganisms	PE, PP and PS	DOC (mg/g): 68.2 ± 0.9 (ePS); 39.1 ± 0.3 (PP); 4.8 ± 0.1 (PEstd); 1.1 ± 0.2 (PE)	enhanced/ reduced growth	DOC bioavailability (%): 76 ± 8 (ePS); 59 ± 8 (PP); 46 ± 8 (PE); 22 ± 4 (PEstd)	77
Planktonic microorganisms	PE	18.6% labile (DOC)	enhanced growth	bacteria protein production (μgC/L/hr): 0.058–0.105 (control) vs 0.132–0.240<br>bacterial growth efficiency (%):5.8–11.5 (control) vs 10.0–19.5	78
Planktonic microorganisms	PE, PP	DOC ($\mu g/cm^2$): 8.92 vs 6.67 (LDPE); 2.79 vs 6.28 (HDPE); 0.26 vs 0.31 (PE packaging); 1.61 vs 2.17 (PP packaging)	enhanced growth	DOC consumption (μM/L): 150.4 vs 92.1 (LDPE); 69.6 vs 136.3 (HDPE); 30.9 vs 27.2 (PE packaging); 52.5 vs 55 (PP packaging);<br>Leucine incorporation (nM/L/h): 0.9 vs 2.5 (LDPE); 0.4 vs 2.5 (HDPE); 1.6 vs 3.3 (PE packaging); 1.3 vs 2.6 (PP packaging);	79

Note. F0 and F1 mean the parent generation and the first generation of offspring; OD600 and OD595 mean optical density at 600 and 595 nm representing cell concentration; OUT number represents the abundance of species; Shannon index represents the diversity of species; Chao 1 index represents species richness

REFERENCE

1. Muller, A.; Becker, R.; Dorgerloh, U.; Simon, F. G.; Braun, U., The effect of polymer aging on the uptake of fuel aromatics and ethers by microplastics. *Environ Pollut* **2018,** *240*, 639-646.
2. Lin, J.; Yan, D.; Fu, J.; Chen, Y.; Ou, H., Ultraviolet-C and vacuum ultraviolet inducing surface degradation of microplastics. *Water Research* **2020,** *186*.
3. Liu, G.; Zhu, Z.; Yang, Y.; Sun, Y.; Yu, F.; Ma, J., Sorption behavior and mechanism of hydrophilic organic chemicals to virgin and aged microplastics in freshwater and seawater. *Environ Pollut* **2019,** *246*, 26-33.
4. Fan, X.; Zou, Y.; Geng, N.; Liu, J.; Hou, J.; Li, D.; Yang, C.; Li, Y., Investigation on the adsorption and desorption behaviors of antibiotics by degradable MPs with or without UV ageing process. *J Hazard Mater* **2021,** *401*, 123363.
5. Liu, J.; Zhang, T.; Tian, L.; Liu, X.; Qi, Z.; Ma, Y.; Ji, R.; Chen, W., Aging Significantly Affects Mobility and Contaminant-Mobilizing Ability of Nanoplastics in Saturated Loamy Sand. *Environ Sci Technol* **2019,** *53*, (10), 5805-5815.
6. Jiang, Z.; Huang, L.; Fan, Y.; Zhou, S.; Zou, X., Contrasting effects of microplastic aging upon the adsorption of sulfonamides and its mechanism. *Chemical Engineering Journal* **2022,** *430*.
7. Kim, T. K.; Jang, M.; Hwang, Y. S., Adsorption of benzalkonium chlorides onto polyethylene microplastics: Mechanism and toxicity evaluation. *J Hazard Mater* **2022,** *426*, 128076.
8. Liu, P.; Lu, K.; Li, J.; Wu, X.; Qian, L.; Wang, M.; Gao, S., Effect of aging on adsorption behavior of polystyrene microplastics for pharmaceuticals: Adsorption mechanism and role of aging intermediates. *J Hazard Mater* **2020,** *384*, 121193.
9. Liu, Y.; Huang, Z.; Zhou, J.; Tang, J.; Yang, C.; Chen, C.; Huang, W.; Dang, Z., Influence of environmental and biological macromolecules on aggregation kinetics of nanoplastics in aquatic systems. *Water Res* **2020,** *186*, 116316.
10. Liu, X.; Sun, P.; Qu, G.; Jing, J.; Zhang, T.; Shi, H.; Zhao, Y., Insight into the characteristics and sorption behaviors of aged polystyrene microplastics through three type of accelerated oxidation processes. *J Hazard Mater* **2021,** *407*, 124836.
11. Wang, Y.; Liu, C.; Wang, F.; Sun, Q., Behavior and mechanism of atrazine adsorption on pristine and aged microplastics in the aquatic environment: Kinetic and thermodynamic studies. *Chemosphere* **2022,** *292*, 133425.
12. Wu, J.; Xu, P.; Chen, Q.; Ma, D.; Ge, W.; Jiang, T.; Chai, C., Effects of polymer aging on sorption of 2,2',4,4'-tetrabromodiphenyl ether by polystyrene microplastics. *Chemosphere* **2020,** *253*, 126706.
13. Fu, J.; Li, Y.; Peng, L.; Gao, W.; Wang, G., Distinct chemical adsorption behaviors of sulfanilamide as a model antibiotic onto weathered microplastics in complex systems. *Colloids and Surfaces A: Physicochemical and Engineering Aspects* **2022,** *648*.
14. Xue, X. D.; Fang, C. R.; Zhuang, H. F., Adsorption behaviors of the pristine and aged thermoplastic polyurethane microplastics in Cu(II)-OTC coexisting system. *J Hazard Mater* **2021,** *407*, 124835.
15. Liu, Y.; Hu, Y.; Yang, C.; Chen, C.; Huang, W.; Dang, Z., Aggregation kinetics of UV irradiated nanoplastics in aquatic environments. *Water Res* **2019,** *163*, 114870.
16. Mao, Y.; Li, H.; Huangfu, X.; Liu, Y.; He, Q., Nanoplastics display strong stability in aqueous environments: Insights from aggregation behaviour and theoretical calculations. *Environ Pollut* **2020,** *258*, 113760.
17. Li, X.; Ji, S.; He, E.; Peijnenburg, W.; Cao, X.; Zhao, L.; Xu, X.; Zhang, P.; Qiu, H., UV/ozone induced physicochemical transformations of polystyrene nanoparticles and their

aggregation tendency and kinetics with natural organic matter in aqueous systems. *J Hazard Mater* **2022,** *433*, 128790.
18. Xu, Y.; Ou, Q.; He, Q.; Wu, Z.; Ma, J.; Huangfu, X., Influence of dissolved black carbon on the aggregation and deposition of polystyrene nanoplastics: Comparison with dissolved humic acid. *Water Res* **2021,** *196*, 117054.
19. Xu, Y.; Ou, Q.; Li, X.; Wang, X.; van der Hoek, J. P.; Liu, G., Combined effects of photoaging and natural organic matter on the colloidal stability of nanoplastics in aquatic environments. *Water Research* **2022**.
20. Zhang, Y. N.; Cheng, F.; Zhang, T.; Li, C.; Qu, J.; Chen, J.; Peijnenburg, W., Dissolved Organic Matter Enhanced the Aggregation and Oxidation of Nanoplastics under Simulated Sunlight Irradiation in Water. *Environ Sci Technol* **2022,** *56*, (5), 3085-3095.
21. Wang, X.; Li, Y.; Zhao, J.; Xia, X.; Shi, X.; Duan, J.; Zhang, W., UV-induced aggregation of polystyrene nanoplastics: effects of radicals, surface functional groups and electrolyte. *Environmental Science: Nano* **2020,** *7*, (12), 3914-3926.
22. Li, X.; He, E.; Jiang, K.; Peijnenburg, W.; Qiu, H., The crucial role of a protein corona in determining the aggregation kinetics and colloidal stability of polystyrene nanoplastics. *Water Res* **2021,** *190*, 116742.
23. Yu, S.; Shen, M.; Li, S.; Fu, Y.; Zhang, D.; Liu, H.; Liu, J., Aggregation kinetics of different surface-modified polystyrene nanoparticles in monovalent and divalent electrolytes. *Environ Pollut* **2019,** *255*, (Pt 2), 113302.
24. Wu, X.; Liu, P.; Gong, Z.; Wang, H.; Huang, H.; Shi, Y.; Zhao, X.; Gao, S., Humic Acid and Fulvic Acid Hinder Long-Term Weathering of Microplastics in Lake Water. *Environ Sci Technol* **2021**.
25. Chen, C.; Chen, L.; Yao, Y.; Artigas, F.; Huang, Q.; Zhang, W., Organotin Release from Polyvinyl Chloride Microplastics and Concurrent Photodegradation in Water: Impacts from Salinity, Dissolved Organic Matter, and Light Exposure. *Environ Sci Technol* **2019,** *53*, (18), 10741-10752.
26. Qiu, X.; Ma, S.; Zhang, J.; Fang, L.; Guo, X.; Zhu, L., Dissolved Organic Matter Promotes the Aging Process of Polystyrene Microplastics under Dark and Ultraviolet Light Conditions: The Crucial Role of Reactive Oxygen Species. *Environ Sci Technol* **2022,** *56*, (14), 10149-10160.
27. Bai, X.; Ma, W.; Zhang, Q.; Zhang, L.; Zhong, S.; Shu, X., Photon-induced redox chemistry on pyrite promotes photoaging of polystyrene microplastics. *Sci Total Environ* **2022,** *829*, 154441.
28. Ding, L.; Yu, X.; Guo, X.; Zhang, Y.; Ouyang, Z.; Liu, P.; Zhang, C.; Wang, T.; Jia, H.; Zhu, L., The photodegradation processes and mechanisms of polyvinyl chloride and polyethylene terephthalate microplastic in aquatic environments: Important role of clay minerals. *Water Res* **2022,** *208*, 117879.
29. Ding, L.; Guo, X.; Du, S.; Cui, F.; Zhang, Y.; Liu, P.; Ouyang, Z.; Jia, H.; Zhu, L., Insight into the Photodegradation of Microplastics Boosted by Iron (Hydr)oxides. *Environ Sci Technol* **2022**.
30. Ding, L.; Ouyang, Z.; Liu, P.; Wang, T.; Jia, H.; Guo, X., Photodegradation of microplastics mediated by different types of soil: The effect of soil components. *Sci Total Environ* **2022,** *802*, 149840.
31. Wang, C.; Xian, Z.; Jin, X.; Liang, S.; Chen, Z.; Pan, B.; Wu, B.; Ok, Y. S.; Gu, C., Photo-aging of polyvinyl chloride microplastic in the presence of natural organic acids. *Water Res* **2020,** *183*, 116082.
32. Khaled, A.; Rivaton, A.; Richard, C.; Jaber, F.; Sleiman, M., Phototransformation of Plastic Containing Brominated Flame Retardants: Enhanced Fragmentation and Release of Photoproducts to Water and Air. *Environ Sci Technol* **2018,** *52*, (19), 11123-11131.

33. Liu, P.; Li, H.; Wu, J.; Wu, X.; Shi, Y.; Yang, Z.; Huang, K.; Guo, X.; Gao, S., Polystyrene microplastics accelerated photodegradation of co-existed polypropylene via photosensitization of polymer itself and released organic compounds. *Water Res* **2022,** *214*, 118209.
34. Liu, P.; Wu, X.; Peng, J.; Wang, H.; Shi, Y.; Huang, H.; Gao, S., Critical effect of iron red pigment on photoaging behavior of polypropylene microplastics in artificial seawater. *J Hazard Mater* **2021,** *404*, (Pt B), 124209.
35. Wu, X.; Liu, P.; Shi, H.; Wang, H.; Huang, H.; Shi, Y.; Gao, S., Photo aging and fragmentation of polypropylene food packaging materials in artificial seawater. *Water Res* **2021,** *188*, 116456.
36. Zhu, K.; Sun, Y.; Jiang, W.; Zhang, C.; Dai, Y.; Liu, Z.; Wang, T.; Guo, X.; Jia, H., Inorganic anions influenced the photoaging kinetics and mechanism of polystyrene microplastic under the simulated sunlight: Role of reactive radical species. *Water Res* **2022,** *216*, 118294.
37. Wu, X.; Liu, P.; Wang, H.; Huang, H.; Shi, Y.; Yang, C.; Gao, S., Photo aging of polypropylene microplastics in estuary water and coastal seawater: Important role of chlorine ion. *Water Res* **2021,** *202*, 117396.
38. Yu, X.; Xu, Y.; Lang, M.; Huang, D.; Guo, X.; Zhu, L., New insights on metal ions accelerating the aging behavior of polystyrene microplastics: Effects of different excess reactive oxygen species. *Sci Total Environ* **2022,** *821*, 153457.
39. Tong, L.; Song, K.; Wang, Y.; Yang, J.; Ji, J.; Lu, J.; Chen, Z.; Zhang, W., Zinc oxide nanoparticles dissolution and toxicity enhancement by polystyrene microplastics under sunlight irradiation. *Chemosphere* **2022,** *299*, 134421.
40. Zhang, X.; Su, H.; Gao, P.; Li, B.; Feng, L.; Liu, Y.; Du, Z.; Zhang, L., Effects and mechanisms of aged polystyrene microplastics on the photodegradation of sulfamethoxazole in water under simulated sunlight. *J Hazard Mater* **2022,** *433*, 128813.
41. Chen, M.; Zhao, X.; Wu, D.; Peng, L.; Fan, C.; Zhang, W.; Li, Q.; Ge, C., Addition of biodegradable microplastics alters the quantity and chemodiversity of dissolved organic matter in latosol. *Sci Total Environ* **2022,** *816*, 151960.
42. Huang, J.; Duan, P.; Tong, L.; Zhang, W., Influence of polystyrene microplastics on the volatilization, photodegradation and photoinduced toxicity of anthracene and pyrene in freshwater and artificial seawater. *Sci Total Environ* **2022,** *819*, 152049.
43. Yin, L.; Wu, N.; Qu, R.; Zhu, F.; Ajarem, J. S.; Allam, A. A.; Wang, Z.; Huo, Z., Insight into the photodegradation and universal interactive products of 2,2',4,4'-tetrabromodiphenyl ether on three microplastics. *J Hazard Mater* **2022,** *445*, 130475.
44. Wang, H. J.; Lin, H. H.; Hsieh, M. C.; Lin, A. Y., Photoaged polystyrene microplastics serve as photosensitizers that enhance cimetidine photolysis in an aqueous environment. *Chemosphere* **2022,** *290*, 133352.
45. Wang, H.; Liu, P.; Wang, M.; Wu, X.; Shi, Y.; Huang, H.; Gao, S., Enhanced phototransformation of atorvastatin by polystyrene microplastics: Critical role of aging. *J Hazard Mater* **2021,** *408*, 124756.
46. Wang, C.; Liang, S.; Bai, L.; Gu, X.; Jin, X.; Xian, Z.; Wu, B.; Ok, Y. S.; Li, K.; Wang, R.; Zhong, H.; Gu, C., Structure-dependent surface catalytic degradation of cephalosporin antibiotics on the aged polyvinyl chloride microplastics. *Water Res* **2021,** *206*, 117732.
47. Ding, R.; Ouyang, Z.; Bai, L.; Zuo, X.; Xiao, C.; Guo, X., What are the drivers of tetracycline photolysis induced by polystyrene microplastic? *Chemical Engineering Journal* **2022,** *435*.
48. Chen, C.; Chen, L.; Li, Y.; Fu, W.; Shi, X.; Duan, J.; Zhang, W., Impacts of microplastics on organotins' photodegradation in aquatic environments. *Environ Pollut* **2020,** *267*, 115686.

49. Ouyang, Z.; Li, S.; Xue, J.; Liao, J.; Xiao, C.; Zhang, H.; Li, X.; Liu, P.; Hu, S.; Guo, X.; Zhu, L., Dissolved organic matter derived from biodegradable microplastic promotes photo-aging of coexisting microplastics and alters microbial metabolism. *Journal of Hazardous Materials* **2023,** *445*.
50. Zhang, P.; Liu, Y.; Zhang, L.; Xu, M.; Gao, L.; Zhao, B., The interaction of micro/nano plastics and the environment: Effects of ecological corona on the toxicity to aquatic organisms. *Ecotoxicol Environ Saf* **2022,** *243*, 113997.
51. Zhang, E.; Chen, Y.; Li, Y.; Sun, K.; Yang, Y.; Gao, B.; Xing, B., The photo-redox of chromium regulated by microplastics (MPs) and MPs-derived dissolved organic matter (MPs-DOM) and the CO2 emission of MPs-DOM. *Fundamental Research* **2022**.
52. Zhang, W.; Song, K.; Ding, R.; Han, H.; Yao, L.; Ji, M.; Chen, Z.; Yu, H.; Wu, C.; Fang, T., Role of polystyrene microplastics in sunlight-mediated transformation of silver in aquatic environments: Mechanisms, kinetics and toxicity. *J Hazard Mater* **2021,** *419*, 126429.
53. Huang, Y.; Dang, F.; Yin, Y.; Fang, G.; Wang, Y.; Yu, G.; Zhou, D.; Xing, B., Weathered Microplastics Induce Silver Nanoparticle Formation. *Environmental Science & Technology Letters* **2021,** *9*, (2), 179-185.
54. Gao, Z.; Chou, P. I.; Liu, J.; Zhu, Y.; Jun, Y. S., Oxidative Roles of Polystyrene-Based Nanoplastics in Inducing Manganese Oxide Formation under Light Illumination. *ACS Nano* **2022**.
55. Tong, L.; Duan, P.; Tian, X.; Huang, J.; Ji, J.; Chen, Z.; Yang, J.; Yu, H.; Zhang, W., Polystyrene microplastics sunlight-induce oxidative dissolution, chemical transformation and toxicity enhancement of silver nanoparticles. *Sci Total Environ* **2022,** *827*, 154180.
56. Zou, W.; Xia, M.; Jiang, K.; Cao, Z.; Zhang, X.; Hu, X., Photo-Oxidative Degradation Mitigated the Developmental Toxicity of Polyamide Microplastics to Zebrafish Larvae by Modulating Macrophage-Triggered Proinflammatory Responses and Apoptosis. *Environ Sci Technol* **2020,** *54*, (21), 13888-13898.
57. Zhang, X.; Zhao, J.; Gan, T.; Jin, C.; Li, X.; Cao, Z.; Jiang, K.; Zou, W., Aging relieves the promotion effects of polyamide microplastics on parental transfer and developmental toxicity of TDCIPP to zebrafish offspring. *J Hazard Mater* **2022,** *437*, 129409.
58. Zhang, X.; Xia, M.; Su, X.; Yuan, P.; Li, X.; Zhou, C.; Wan, Z.; Zou, W., Photolytic degradation elevated the toxicity of polylactic acid microplastics to developing zebrafish by triggering mitochondrial dysfunction and apoptosis. *J Hazard Mater* **2021,** *413*, 125321.
59. Zhang, X.; Xia, M.; Zhao, J.; Cao, Z.; Zou, W.; Zhou, Q., Photoaging enhanced the adverse effects of polyamide microplastics on the growth, intestinal health, and lipid absorption in developing zebrafish. *Environ Int* **2022,** *158*, 106922.
60. Zhang, P.; Lu, G.; Sun, Y.; Yan, Z.; Dang, T.; Liu, J., Metagenomic analysis explores the interaction of aged microplastics and roxithromycin on gut microbiota and antibiotic resistance genes of Carassius auratus. *J Hazard Mater* **2022,** *425*, 127773.
61. Wang, X.; Zheng, H.; Zhao, J.; Luo, X.; Wang, Z.; Xing, B., Photodegradation Elevated the Toxicity of Polystyrene Microplastics to Grouper (Epinephelus moara) through Disrupting Hepatic Lipid Homeostasis. *Environ Sci Technol* **2020,** *54*, (10), 6202-6212.
62. Huang, Y.; Ding, J.; Zhang, G.; Liu, S.; Zou, H.; Wang, Z.; Zhu, W.; Geng, J., Interactive effects of microplastics and selected pharmaceuticals on red tilapia: Role of microplastic aging. *Sci Total Environ* **2021,** *752*, 142256.
63. Wang, Z.; Fu, D.; Gao, L.; Qi, H.; Su, Y.; Peng, L., Aged microplastics decrease the bioavailability of coexisting heavy metals to microalga Chlorella vulgaris. *Ecotoxicol Environ Saf* **2021,** *217*, 112199.
64. Wang, Q.; Wangjin, X.; Zhang, Y.; Wang, N.; Wang, Y.; Meng, G.; Chen, Y., The toxicity of virgin and UV-aged PVC microplastics on the growth of freshwater algae Chlamydomonas reinhardtii. *Sci Total Environ* **2020,** *749*, 141603.

65. Chen, H.; Yang, Y.; Wang, C.; Hua, X.; Li, H.; Xie, D.; Xiang, M.; Yu, Y., Reproductive toxicity of UV-photodegraded polystyrene microplastics induced by DNA damage-dependent cell apoptosis in Caenorhabditis elegans. *Sci Total Environ* **2022,** *811*, 152350.
66. Giri, S.; Mukherjee, A., Ageing with algal EPS reduces the toxic effects of polystyrene nanoplastics in freshwater microalgae Scenedesmus obliquus. *Journal of Environmental Chemical Engineering* **2021,** *9*, (5).
67. Li, Z.; Xie, Y.; Zeng, Y.; Zhang, Z.; Song, Y.; Hong, Z.; Ma, L.; He, M.; Ma, H.; Cui, F., Plastic leachates lead to long-term toxicity in fungi and promote biodegradation of heterocyclic dye. *Sci Total Environ* **2022,** *806*, (Pt 1), 150538.
68. Zhao, C.; Xu, T.; He, M.; Shah, K. J.; You, Z.; Zhang, T.; Zubair, M., Exploring the toxicity of the aged styrene-butadiene rubber microplastics to petroleum hydrocarbon-degrading bacteria under compound pollution system. *Ecotoxicol Environ Saf* **2021,** *227*, 112903.
69. Bao, R.; Cheng, Z.; Hou, Y.; Xie, C.; Pu, J.; Peng, L.; Gao, L.; Chen, W.; Su, Y., Secondary microplastics formation and colonized microorganisms on the surface of conventional and degradable plastic granules during long-term UV aging in various environmental media. *Journal of Hazardous Materials* **2022,** *439*.
70. Gong, M.; Yang, G.; Zhuang, L.; Zeng, E. Y., Microbial biofilm formation and community structure on low-density polyethylene microparticles in lake water microcosms. *Environ Pollut* **2019,** *252*, (Pt A), 94-102.
71. Shan, E.; Zhang, X.; Li, J.; Sun, C.; Teng, J.; Yang, X.; Chen, L.; Liu, Y.; Sun, X.; Zhao, J.; Wang, Q., Incubation habitats and aging treatments affect the formation of biofilms on polypropylene microplastics. *Sci Total Environ* **2022,** *831*, 154769.
72. Chen, M.; Liu, S.; Bi, M.; Yang, X.; Deng, R.; Chen, Y., Aging behavior of microplastics affected DOM in riparian sediments: From the characteristics to bioavailability. *J Hazard Mater* **2022,** *431*, 128522.
73. Liu, Y.; Zhou, H.; Yan, M.; Liu, Y.; Ni, X.; Song, J.; Yi, X., Toxicity of tire wear particles and the leachates to microorganisms in marine sediments. *Environ Pollut* **2022,** *309*, 119744.
74. Yu, Y.; Li, X.; Feng, Z.; Xiao, M.; Ge, T.; Li, Y.; Yao, H., Polyethylene microplastics alter the microbial functional gene abundances and increase nitrous oxide emissions from paddy soils. *J Hazard Mater* **2022,** *432*, 128721.
75. Zhang, Y.; Li, X.; Xiao, M.; Feng, Z.; Yu, Y.; Yao, H., Effects of microplastics on soil carbon dioxide emissions and the microbial functional genes involved in organic carbon decomposition in agricultural soil. *Sci Total Environ* **2022,** *806*, (Pt 3), 150714.
76. Choi, H.-J.; Ju, W. J.; An, J., Impact of the Virgin and Aged Polystyrene and Polypropylene Microfibers on the Soil Enzyme Activity and the Microbial Community Structure. *Water, Air, & Soil Pollution* **2021,** *232*, (8).
77. Zhu, L.; Zhao, S.; Bittar, T. B.; Stubbins, A.; Li, D., Photochemical dissolution of buoyant microplastics to dissolved organic carbon: Rates and microbial impacts. *J Hazard Mater* **2020,** *383*, 121065.
78. Sheridan, E. A.; Fonvielle, J. A.; Cottingham, S.; Zhang, Y.; Dittmar, T.; Aldridge, D. C.; Tanentzap, A. J., Plastic pollution fosters more microbial growth in lakes than natural organic matter. *Nat Commun* **2022,** *13*, (1), 4175.
79. Romera-Castillo, C.; Pinto, M.; Langer, T. M.; Alvarez-Salgado, X. A.; Herndl, G. J., Dissolved organic carbon leaching from plastics stimulates microbial activity in the ocean. *Nat Commun* **2018,** *9*, (1), 1430.
